# Convergence in Pruning and Circadian Regulation in Obsessive-Compulsive Disorder and Allergic Diseases: Evidence From a Secondary Genome-Wide Analysis

**DOI:** 10.7759/cureus.109781

**Published:** 2026-05-27

**Authors:** Ngo Cheung

**Affiliations:** 1 Psychiatry, Cheung Ngo Medical Limited, Hong Kong, HKG

**Keywords:** allergic diseases, allergy, circadian regulation, eczema, genome-wide analysis, obsessive-compulsive disorder, ocd, ocd and related disorder, pruning, twas

## Abstract

Background: Obsessive-compulsive disorder (OCD) and allergic diseases co-occur far more often than expected, yet genome-wide genetic correlations are typically modest or negative. The objective of this study was to test whether OCD and allergic diseases show convergence at the level of pre-specified biological pathways, especially synaptic pruning and circadian regulation, rather than at the level of broad genome-wide correlation.

Methods: Using the Multi-marker Analysis of GenoMic Annotation (MAGMA) pipeline, we re-analysed summary statistics from a large allergic-diseases genome-wide association study (GWAS) (N = 360,838). We then used gene set enrichment analysis (GSEA) and cross-tissue transcriptome-wide association study (TWAS) analyses with S-PrediXcan and Genotype-Tissue Expression (GTEx) version 8 prediction models.

Results: The synaptic pruning gene set reached Bonferroni-corrected significance in allergic diseases (p = 6.75 × 10⁻⁵), driven by major histocompatibility complex (MHC) class I genes (HLA-B, HLA-A, HLA-C, TAP2), complement genes (C4A, C4B), and inflammatory regulators (NFKB1, RELA). The glutamatergic signalling set also survived the eight-set Bonferroni threshold (p = 0.005). The REACTOME Circadian Clock set showed supportive enrichment in the MAGMA analysis (raw p = 1.92 × 10⁻³; Bonferroni-adjusted p = 0.015), although this did not meet the pre-specified eight-set Bonferroni threshold of 0.0063. Standard GSEA nevertheless showed REACTOME Circadian Clock enrichment in both allergic diseases and OCD. Cross-tissue TWAS projected allergic risk onto six OCD-relevant brain regions and yielded false discovery rate (FDR)-significant pruning and circadian hits, including TAP2 in amygdala (p = 3.02 × 10⁻¹¹). Projecting OCD risk onto allergy-relevant peripheral tissues produced strong circadian gene-set enrichment, especially for the Kyoto Encyclopedia of Genes and Genomes (KEGG) Circadian Rhythm Mammal set (1.95-fold, p = 1.62 × 10⁻⁸). Integration with published atopic dermatitis transcriptomic studies, rather than new skin transcriptomic profiling in this study, suggested global clock downregulation alongside specific shifts involving ARNTL2, NOCT, and RORC.

Conclusions: These findings support pathway-level and candidate gene-level convergence between OCD and allergic diseases, centred on pruning biology and circadian regulation. The proposed skin-brain neuroimmune loop is a hypothetical framework, not causal proof, and requires functional and longitudinal validation. The results move beyond broad polygenic correlations and identify immune-circadian pathways that may be worth testing in future mechanistic and clinical studies of comorbid patients.

## Introduction

Obsessive-compulsive disorder and its burden

Obsessive-compulsive disorder (OCD) is a long-lasting and often disabling neuropsychiatric disorder. Obsessive thoughts that make people anxious and compulsive behaviours or mental acts that people do to feel better are what define it. Estimates suggest that roughly 1% to 3% of the global population is affected across the lifespan, placing OCD among the top 10 causes of disability worldwide and imposing considerable personal, social, and economic costs [1,2]. Treatments that work do exist, including exposure and response prevention therapy and serotonin reuptake inhibitors, but somewhere between 40% and 60% of patients achieve only partial remission or remain resistant to treatment altogether. This reality highlights how much we still need to learn about what drives the disorder.

For many years, researchers focused primarily on dysfunction within cortico-striato-thalamo-cortical circuits. In this model, orbitofrontal and anterior cingulate cortical regions interact with the caudate, putamen, globus pallidus, thalamus, and related limbic structures to regulate error monitoring, threat appraisal, response inhibition, habit formation, and action selection. Overactivity or inefficient gating within these loops is thought to leave intrusive thoughts and repetitive behaviours insufficiently filtered, which helps explain why compulsions can persist even when patients recognise them as excessive [2,3]. More recently, though, a growing body of evidence has drawn attention to how often OCD co-occurs with somatic conditions, especially those that involve immune dysregulation. Allergic diseases, including atopic dermatitis, allergic rhinitis, asthma, and severe manifestations like anaphylaxis, have stood out as especially common companions to OCD.

In this study, allergic diseases refer mainly to the broad atopic phenotype used in the source genome-wide association study (GWAS): asthma, hay fever or allergic rhinitis, and eczema or atopic dermatitis. These conditions differ clinically, but they share a tendency towards epithelial-barrier dysfunction, type 2 helper T-cell inflammation, immunoglobulin E-mediated sensitisation, and recurrent interaction between environmental allergens and mucosal or skin surfaces [4,5].

Epidemiological evidence linking OCD and allergic diseases

Several large-scale epidemiological studies have now measured this comorbidity with notable precision. A Swedish retrospective population-based case-control analysis, which drew on data from nearly 400,000 individuals, found that patients diagnosed with atopic dermatitis had roughly double the odds of also receiving an OCD diagnosis when compared to matched controls (adjusted odds ratio = 2.0, 95% confidence interval = 1.5 to 2.7, p less than 0.001) [6]. This link held up even after the researchers controlled for other atopic conditions that might have muddied the picture. More recently, a large UK retrospective cohort study reinforced these findings in a big way. Working with data from over 5.6 million primary care patients, the authors found that people with documented atopic disorders had a 20% greater hazard of developing mental health problems in general, and OCD stood out in particular (adjusted hazard ratio = 1.20, 95% confidence interval = 1.14 to 1.26) [7]. What really caught attention, though, was how steeply the risk rose with more severe allergic presentations. Anaphylaxis, for instance, carried an adjusted hazard ratio of 2.37. Earlier general-population work had similarly linked obsessive-compulsive symptoms to higher rates of allergies (odds ratio = 1.6), migraine (odds ratio = 1.9), and respiratory conditions (odds ratio = 1.7), with the combination of physical and psychiatric comorbidity tied to a greater number of disability days [8]. These patterns appear especially pronounced among children and individuals with early-onset presentations, where the burden of allergic disease may interact with ongoing neurodevelopment. There are also hints of bidirectionality: compulsive washing behaviours, for instance, may worsen skin barrier dysfunction, while chronic itch and inflammation could reinforce obsessive thought patterns.

The genetic landscape and the gap between phenotype and genotype

Even with these fairly solid phenotypic links on the table, we are still largely in the dark about what connects OCD and allergic disease at a biological level; most of it has been educated speculation. On the OCD side, though, the genetics have moved fast. A major genome-wide association meta-analysis identified 30 independent risk loci and established that the disorder has considerable polygenic heritability [9]. Building on that, follow-up studies using several different analytical approaches have zeroed in on dysregulated synaptic pruning as a key piece of the puzzle, specifically, complement-mediated microglial activity and oligodendroglial support gone awry [10].

Synaptic pruning is the developmental process through which excess or weak synaptic connections are removed while stronger and more useful connections are retained. In the central nervous system, this process is not simply a neuronal housekeeping event: complement proteins such as C1q and C3 can tag synapses, and microglia can then engulf tagged synaptic material. When this system is mistimed or excessive, circuit refinement can plausibly become maladaptive rather than adaptive [11]. Those same OCD analyses flagged circadian clock genes as potential temporal gatekeepers, possibly shaping when during development the brain is most vulnerable to this kind of aberrant pruning.

Circadian clock genes are the molecular components of the roughly 24-hour timing system that coordinates sleep-wake behaviour, immune activity, metabolism, hormone release, and tissue repair. In mammals, CLOCK and BMAL1 activate transcription of period and cryptochrome genes, while PER and CRY proteins feed back to inhibit their own transcription; REV-ERB and ROR nuclear receptors add another regulatory loop around BMAL1 and related clock outputs [12]. In practical terms, these genes do not just keep time in the brain; they also regulate peripheral tissues, including skin, lung, blood, liver, adipose tissue, and immune cells.

On the allergy side of things, the landmark GWAS by Ferreira et al. demonstrated that asthma, hay fever, and eczema share a striking amount of genetic liability, with much of it clustered in loci tied to immune regulation, particularly Th2 signalling and epithelial-barrier pathways [5]. Yet when researchers have looked at genome-wide genetic correlations between allergic traits and psychiatric disorders, including OCD, the overlap has tended to be modest or even negative at the broad single-nucleotide polymorphism level, particularly when certain autoimmune or inflammatory proxies are used. This discrepancy - strong epidemiological comorbidity sitting alongside relatively weak overall genetic overlap - suggests that the most important insights may lie not in pan-genomic pleiotropy but at the level of specific biological pathways.

Gaps in the literature and the present study

Up to this point, no study has directly compared the same curated biological pathways across OCD and allergic-disease genome-wide association data using an identical analytical framework. Furthermore, potential tissue-specific crosstalk, and in particular the possibility that peripheral allergic inflammation and clock-gene dysregulation in the skin might influence central brain circuits relevant to OCD, has received virtually no systematic attention. This is biologically plausible because skin is both an immune organ and a sensory-neural interface. Cutaneous cytokines can drive itch and pain signalling, while sensory neurons can also regulate local immune-cell behaviour, creating a two-way neuroimmune system rather than a passive barrier alone [13]. Such peripheral-to-central interactions could represent a missing mechanistic link, one in which local immune activation amplifies genetically encoded vulnerabilities in how cortico-striato-thalamo-cortical circuits mature.

The present study was designed to address these gaps. We re-analysed summary statistics from a large allergic-diseases GWAS (N = 360,838) using the same Multi-marker Analysis of GenoMic Annotation (MAGMA) gene-set enrichment pipeline that had previously been applied to the 2025 OCD GWAS. We also carried out cross-tissue transcriptome-wide association studies to examine how genetic risk for one condition manifests in tissues relevant to the other - projecting allergic risk onto brain tissues and OCD risk onto skin and other peripheral tissues. Finally, we integrated these genetic findings with existing transcriptomic data on circadian clock-gene expression in atopic-dermatitis skin. Through this multi-layered approach, we identify convergent enrichment in synaptic pruning and circadian regulation pathways and propose a detailed, gene-level mechanistic model, a hypothetical one, that accounts for the observed comorbidity. By connecting peripheral skin biology to central neurodevelopmental processes, this work offers a fresh perspective on the shared origins of OCD and allergic diseases and points towards new avenues for prevention and treatment in patients who live with both.

The specific objectives were as follows: first, to test whether pre-selected pruning, glutamatergic, circadian, monoaminergic, and housekeeping gene sets show enrichment in allergic-disease GWAS data using the same analytic framework previously applied to OCD; second, to examine whether allergic-disease genetic risk maps onto pruning and clock-gene expression in OCD-relevant brain tissues, and whether OCD genetic risk maps onto clock-gene expression in allergy-relevant peripheral tissues.

## Materials and methods

Study design, setting, and ethics

This was a secondary, hypothesis-driven, cross-trait bioinformatic analysis of publicly available or previously published GWAS summary statistics. It combined gene-based association testing, competitive gene-set enrichment, standard gene set enrichment analysis (GSEA), differential GSEA, and cross-tissue transcriptome-wide association study (TWAS). No patient recruitment, clinical intervention, or direct biological sampling was performed for the present analysis. Therefore, no recruitment period in years or months applies to this study; the relevant participant ascertainment windows are those of the original GWAS cohorts [5,9].

Because only de-identified summary statistics and public prediction models were used, ethics approval was not required for this secondary analysis. The original OCD and allergic-disease GWAS studies obtained ethics approvals and participant consent according to their own protocols [5,9].

Overview and data sources

Every analysis reported here followed the same pipeline we had previously used for the 2025 OCD genome-wide association meta-analysis [9,10], so that results from the two traits could be compared on equal footing. Summary statistics for allergic diseases, defined as a combined phenotype of asthma, hay fever, and eczema, came from the European Bioinformatics Institute (EBI) GWAS Catalog (study accession ebi-a-GCST005038) [5]. That dataset included 360,838 individuals of European ancestry, split roughly evenly between 180,129 cases and 180,709 controls. Cases in the source allergic-disease GWAS were individuals coded as having at least one of the following conditions: asthma, hay fever or allergic rhinitis, or eczema or atopic dermatitis; controls were coded as not having these conditions according to the original cohort-level phenotype definitions [5]. The public summary-statistics file did not provide individual-level demographic variables, country-level ancestry assignments, or clinical severity measures.

For OCD, we drew on the 2025 meta-analysis summary statistics [9]. The published source GWAS included 53,660 OCD cases and 2,044,417 controls from 28 case-control cohorts of European ancestry. Case ascertainment varied across cohorts: some cases were diagnosed by a healthcare professional, some were derived from health records or biobanks, and some were based on self-reported OCD diagnosis in consumer or population cohorts. The source study reported 20,427 cases meeting the Diagnostic and Statistical Manual of Mental Disorders, Fifth Edition (DSM-5) or the International Classification of Diseases, Tenth Revision (ICD-10) criteria or health-record-derived criteria and 32,233 self-reported cases [9]. The harmonised summary file used in the present pipeline carried an effective sample-size parameter of 68,099; this value is an analysis parameter and should not be read as the number of OCD cases. The complete available summary statistics files were used; no additional sampling technique was applied.

Both main GWAS datasets were restricted to individuals of European ancestry as defined in their original studies. This reduced, but did not remove, ancestry-related heterogeneity. No trans-ancestry meta-analysis was attempted. The source summary statistics did not contain enough individual-level information to reassign ancestry or model finer sub-European structure directly. Linkage disequilibrium was therefore matched to a European reference panel, and ancestry-related uncertainty is treated as a limitation rather than as a fully solved issue.

MAGMA gene-based and gene-set analysis

We prepared the allergic-disease summary statistics for gene-based testing with MAGMA version 1.10 [14]. The VCF (Variant Call Format) file was first converted into the single nucleotide polymorphism (SNP) location and p-value format that MAGMA requires. After dropping 119,922 non-autosomal SNPs and another 999 whose p-values were missing or otherwise unusable, 7,996,580 autosomal SNPs remained for analysis.

Because the present analysis used summary statistics rather than individual genotype data, individual-level quality control could not be re-run. Strand alignment, imputation quality filtering, removal of duplicate variants, relatedness filtering, and cohort-level allele harmonisation were inherited from the source GWAS pipelines [5,9]. In the present pipeline, variants with missing or invalid p-values and non-autosomal variants were removed. No additional minor allele frequency filter was imposed at this stage beyond the filters already used by the source GWAS and the availability of variants in the European linkage disequilibrium reference.

Genes were annotated using the National Center for Biotechnology Information (NCBI) Build 37.3 (hg19) coordinates, with windows extending 35 kb upstream and 10 kb downstream of each gene body. This window was selected to match the earlier OCD pipeline exactly, rather than to optimise the allergic-disease results after seeing them. The aim was to keep gene mapping comparable across traits. Linkage disequilibrium was estimated from the European panel of the 1000 Genomes Project Phase 3. The full 1000 Genomes Phase 3 resource contains 2,504 individuals from 26 populations; the European reference panel used for linkage disequilibrium estimation contains 503 individuals from CEU (Utah Residents with Northern and Western European Ancestry), FIN (Finnish in Finland), GBR (British from England and Scotland), IBS (Iberian Populations in Spain), and TSI (Tuscans from Italy) populations [15]. The gene-level test statistic was the mean chi-square, weighted by linkage disequilibrium and adjusted for the effective sample size of 360,838. In all, 18,270 protein-coding genes were tested.

MAGMA commands followed the standard sequence: annotation with the 35 kb upstream and 10 kb downstream window, gene analysis using the 1000 Genomes European reference panel, and competitive gene-set testing against the full tested gene background. The core settings corresponded to --annotate window=35,10, --bfile using the 1000 Genomes European reference, --pval with the allergy p-value file and N = 360838, --gene-analysis --model mean, and --gene-set for competitive enrichment.

We then ran competitive gene-set enrichment tests on eight sets chosen ahead of time. These comprised a curated synaptic pruning set containing 211 genes, a glutamatergic signalling set of 121 genes, four circadian clock-related sets (REACTOME Circadian Clock with 111 genes; Kyoto Encyclopedia of Genes and Genomes (KEGG) Circadian Rhythm Mammal with 13 genes; WikiPathways (WP) Circadian Rhythm Genes with 201 genes; and Gene Ontology Biological Process (GOBP) Regulation of Circadian Rhythm with 119 genes), and two negative-control sets - one for housekeeping genes (182 genes) and one for the monoamine pathway (101 genes). We corrected p-values across the eight tests using the Bonferroni method, setting the significance threshold at 0.0063.

The synaptic pruning gene set was curated before analysing the allergic-disease GWAS and was inherited from the previous OCD analysis. Genes were included if they belonged to one or more pruning-relevant biological categories: major histocompatibility complex (MHC) and antigen-presentation machinery, complement tagging, microglial phagocytosis and autophagy, inflammatory signalling, oligodendroglial stabilisation, cytoskeletal remodelling, and axon-guidance or synaptic refinement pathways. Gene symbols were harmonised to HUGO Gene Nomenclature Committee (HGNC)-style symbols where possible, and the same symbol list was used across the MAGMA, GSEA, and TWAS steps. Public circadian sets were taken from MSigDB-linked pathway definitions, including REACTOME, KEGG, WikiPathways, and Gene Ontology Biological Process collections [16-21].

Standard GSEA and differential GSEA

To look at pathway patterns more broadly and to pick out sets that were enriched to different degrees in allergic diseases versus OCD, we carried out a standard gene-set enrichment analysis following the approach of Subramanian et al. [16], running 1,000 phenotype permutations. Alongside this, we performed an exhaustive differential gene-set enrichment analysis comparing the two traits directly. A total of 39 gene sets went into these analyses. They included the primary curated sets already described, standardised public versions of those sets, glial cell-type marker sets for adult microglia, astrocytes, and oligodendrocytes, and the same negative controls used in the MAGMA step. This design lets us identify both shared and trait-specific pathway signals while accounting for differences in background polygenic architecture.

GSEA results were interpreted using permutation p-values and false discovery rate (FDR) values where available. Differential GSEA was treated as exploratory because the number of gene sets was larger and pathway overlap was unavoidable. Full standard GSEA and differential GSEA outputs for the 39 tested sets are provided in the Appendices.

Transcriptome-wide association study with S-PrediXcan

We used S-PrediXcan [22] together with GTEx version 8 MASHR prediction models to impute genetically regulated gene expression [23,24]. MASHR refers to multivariate adaptive shrinkage in R, a modelling approach used in the GTEx v8 prediction weights. The analysis had two complementary arms. In the first, we took the genetic risk profile for allergic diseases and projected it onto six brain tissues that are central to OCD neurocircuitry: frontal cortex (Brodmann area 9), hippocampus, amygdala, anterior cingulate cortex (Brodmann area 24), nucleus accumbens, and caudate. These regions were selected because they map onto error monitoring, affective salience, habit learning, and cortico-striatal action gating, which are repeatedly implicated in OCD neurobiology [2,3]. In the second, we took the genetic risk profile for OCD and projected it onto seven tissues relevant to allergic disease and skin barrier function: non-sun-exposed suprapubic skin, sun-exposed lower leg skin, lung, whole blood, subcutaneous adipose tissue, liver, and spleen.

The S-PrediXcan analyses used the MetaXcan/S-PrediXcan framework described by Barbeira et al. and GTEx v8 MASHR models from PredictDB/Zenodo resources [22-24]. The number of genes successfully tested varied by tissue because prediction models and overlapping GWAS variants differ across tissues. In the OCD-to-peripheral-tissue analysis, 12,060 genes were tested in non-sun-exposed skin, 12,325 in sun-exposed skin, 12,037 in the lung, 10,111 in whole blood, 11,863 in subcutaneous adipose tissue, 9,707 in liver, and 10,989 in spleen. In the allergy-to-brain analysis, 11,861 genes were tested in the frontal cortex, 11,357 in the hippocampus, 10,605 in the amygdala, 11,295 in the anterior cingulate cortex, 11,835 in the nucleus accumbens, and 11,907 in the caudate.

Within each tissue, we tested whether our hypothesis-driven gene sets showed stronger association signals than the genome-wide background. We did this by comparing the distribution of absolute Z-scores for genes inside each set against all remaining genes, using one-sided Mann-Whitney U tests. Absolute Z-scores were used to measure the strength of predicted-expression association regardless of direction; positive and negative TWAS Z-scores were therefore both treated as evidence of association strength rather than as directly comparable upregulation or downregulation. Individual gene-tissue associations were considered FDR-significant at Benjamini-Hochberg q < 0.05. Mann-Whitney U enrichment tests were interpreted at p < 0.05, but these gene-set TWAS enrichment results should be viewed as exploratory unless they survived very strong correction or were consistent across related analyses. All analyses were carried out on the hg19 build so that they would be consistent with the reference panels and with the earlier OCD pathway work [10]. Negative-control sets were included at every stage to confirm that any enrichments we observed were specific rather than artefactual.

Data and code availability

All GWAS and prediction-model inputs used here are available from the cited source studies or public repositories: the allergic-disease GWAS from the EBI GWAS Catalog under accession ebi-a-GCST005038, the OCD GWAS from the published 2025 meta-analysis resources, and GTEx v8 prediction models from PredictDB/Zenodo. The custom conversion and summary scripts used for this analysis were not deposited in a public repository at the time of writing and are available from the author on reasonable request.

## Results

Gene-based association testing

We began by running MAGMA gene-based association tests on the allergic diseases GWAS summary statistics (effective N = 360,838) [5]. Following quality control and gene annotation, using a 35 kb upstream and 10 kb downstream window together with the European 1000 Genomes Phase 3 linkage disequilibrium reference, a total of 18,270 protein-coding genes were available for testing. Of these, 239 survived Bonferroni correction for multiple comparisons (p less than 2.74 times 10 to the negative 6). A further 794 genes fell in the suggestive range (p less than 0.001), and 3,025 reached nominal significance (p less than 0.05).

The strongest hits landed squarely in well-known immune and epithelial loci. CLEC16A on chromosome 16 topped the list (p = 2.39 × 10⁻¹⁸), closely followed by IL1R1 on chromosome 2 (p = 2.57 × 10⁻¹⁸) and CAMK4 on chromosome 5 (p = 7.50 × 10⁻¹⁷). Other genes among the top results included IL18R1, IL2RA, TLR10, IL13, HLA-DRA, BTNL2, SMAD3, and IL33 (Table 1). Broadly speaking, these findings lined up with what is already understood about the Th2-driven and barrier-related biology of allergic disease.

**Table 1:** Top 10 genome-wide significant genes in allergic diseases GWAS (MAGMA gene-based analysis). GWAS = genome-wide association study; MAGMA = Multi-marker Analysis of GenoMic Annotation; Chr = chromosome; NSNPs = number of single-nucleotide polymorphisms mapped to the gene; ZSTAT = MAGMA gene-based Z statistic; MHC = major histocompatibility complex. The P-value column gives the uncorrected MAGMA gene-based p-value, and the Bonferroni p gives the p-value adjusted across 18,270 tested protein-coding genes. The genome-wide Bonferroni significance threshold was p < 2.74 × 10⁻⁶.

Rank	Gene symbol	Chr	NSNPs	ZSTAT	P-value	Bonferroni p	Functional category	Relevant target gene-set overlap
1	CLEC16A	16	761	8.6584	2.39 × 10⁻¹⁸	4.37 × 10⁻¹⁴	Immune regulation/autophagy-related locus	None of the primary target sets
2	IL1R1	2	450	8.6502	2.57 × 10⁻¹⁸	4.70 × 10⁻¹⁴	Interleukin-1 signalling/inflammation	Inflammatory-pruning context
3	CAMK4	5	802	8.2565	7.50 × 10⁻¹⁷	1.37 × 10⁻¹²	Calcium/CaMK signalling	Glutamatergics
4	IL18R1	2	315	8.1033	2.67 × 10⁻¹⁶	4.89 × 10⁻¹²	Cytokine receptor signalling	None of the primary target sets
5	IL2RA	10	378	8.0369	4.61 × 10⁻¹⁶	8.42 × 10⁻¹²	T-cell activation/regulation	None of the primary target sets
6	TLR10	4	277	7.9633	8.38 × 10⁻¹⁶	1.53 × 10⁻¹¹	Innate immune receptor	None of the primary target sets
7	IL13	5	74	7.8910	1.50 × 10⁻¹⁵	2.74 × 10⁻¹¹	Type 2 helper T-cell cytokine	Peripheral inflammatory module
8	HLA-DRA	6	443	7.8868	1.55 × 10⁻¹⁵	2.83 × 10⁻¹¹	Major histocompatibility complex class II antigen presentation	MHC-related immune context
9	RERE	1	768	7.8804	1.63 × 10⁻¹⁵	2.98 × 10⁻¹¹	Transcriptional/developmental regulation	None of the primary target sets
10	BTNL2	6	461	7.7713	3.89 × 10⁻¹⁵	7.10 × 10⁻¹¹	MHC-region immune regulation	MHC-related immune context

For comparison, the published 2025 OCD GWAS reported 30 independent genome-wide significant loci and highlighted likely effector genes, including WDR6, DALRD3, CTNND1, and several genes in the MHC region [9]. These OCD gene-level findings were not re-estimated in the present allergic-disease MAGMA run, but they provide the comparator background for the pathway-level analyses below.

Hypothesis-driven gene-set enrichment

We then tested our eight pre-selected gene sets for competitive enrichment, applying the same framework we had previously used with the 2025 OCD GWAS [9,10]. P-values were Bonferroni-corrected across all eight sets, yielding a significance threshold of 0.0063 (Table 2).

**Table 2:** Gene-set enrichment results from MAGMA analysis: allergic diseases GWAS vs. OCD GWAS. MAGMA = Multi-marker Analysis of GenoMic Annotation; GWAS = genome-wide association study; OCD = obsessive-compulsive disorder; REACTOME = Reactome pathway database; KEGG = Kyoto Encyclopedia of Genes and Genomes; WP = WikiPathways; GOBP = Gene Ontology Biological Process. Bonferroni correction was applied across the eight pre-selected tests, giving a significance threshold of 0.0063. NR indicates that the t-statistic was not retained in the exported summary table for non-enriched sets; for sets with Bonferroni-adjusted p = 1.000, the exact raw p-value is bounded at or above 0.125 because of the eight-test Bonferroni correction.

Gene set	Number of genes	Mean Z	t-statistic	Raw p-value	Bonferroni-adjusted p	Significant at p < 0.0063?
Pruning	211	1.1145	4.41	8.43 × 10⁻⁶	6.75 × 10⁻⁵	Yes
Glutamatergics	121	0.979	3.30	6.37 × 10⁻⁴	0.005	Yes
REACTOME Circadian Clock	111	1.009	2.96	1.92 × 10⁻³	0.015	No, supportive only
KEGG Circadian Rhythm Mammal	13	0.784	NR	≥0.125	1.000	No
WP Circadian Rhythm Genes	201	0.625	NR	≥0.125	1.000	No
GOBP Regulation of Circadian Rhythm	119	0.723	NR	Approximately 0.114	0.909	No
Negative Controls: Housekeeping	182	0.632	NR	≥0.125	1.000	No
Monoamines	101	0.549	NR	≥0.125	1.000	No

The synaptic pruning set, which contained 211 genes, was strongly enriched (t = 4.41, uncorrected p = 8.43 × 10⁻⁶, Bonferroni-corrected p = 6.75 × 10⁻⁵). The average Z-score for genes in this set was 1.1145, roughly double the genome-wide mean of 0.564. Within the set, 55 genes reached nominal significance, and 10 cleared the genome-wide threshold. The leading signal came from SMAD3 (Z = 7.61), which sits in the transforming growth factor-beta (TGF-beta)/bone morphogenetic protein (BMP) signalling category. Strong contributions also came from MHC class I molecules (HLA-B, HLA-A, HLA-C, TAP2), complement genes (C4A, C4B), and inflammatory modulators (NFKB1, RELA, TNF).

The glutamatergic signalling set (121 genes) likewise survived correction (t = 3.30, uncorrected p = 6.37 × 10⁻⁴, Bonferroni-corrected p = 0.005), with a mean Z of 0.979. CAMK4, from the CaMKII signalling category, drove much of this signal. The REACTOME_CIRCADIAN_CLOCK set showed supportive enrichment (t = 2.96, uncorrected p = 1.92 × 10⁻³, Bonferroni-corrected p = 0.015; mean Z = 1.009), led by PSMD3 (Z = 7.65), but it did not meet the pre-specified eight-set Bonferroni threshold of 0.0063. The two negative-control sets - housekeeping genes and monoamine pathway genes - showed no hint of enrichment after correction. Notably, the pruning pattern closely mirrored what had been found when the identical analysis was applied to the OCD GWAS [10], while the circadian signal was strongest when the broader GSEA and cross-tissue TWAS layers were considered together.

Standard GSEA and differential GSEA

To get a broader view of pathway-level patterns, and particularly to identify sets that differed in their degree of enrichment between allergic diseases and OCD, we ran both a standard gene-set enrichment analysis with 1,000 phenotype permutations and an exhaustive differential GSEA comparing the two traits across 39 gene sets.

In the standard analysis, the REACTOME Circadian Clock set came through as significantly enriched in both conditions, with a normalised enrichment score of 1.52 (p = 0.001; FDR = 0.017) in allergic diseases and 1.44 (p = 0.009; FDR = 0.045) in OCD. The GOBP Regulation of Circadian Rhythm set was especially prominent in OCD (normalised enrichment score = 1.61, p = 0.001; FDR = 0.009). The allergic-disease analysis also showed enrichment of the clonal haematopoiesis set (normalised enrichment score = 1.49, p = 0.008), while the OCD analysis showed depletion of adult astrocyte markers (normalised enrichment score = -1.59, p = 0.008). The differential analysis revealed that most circadian and pruning-related sets showed modest differential enrichment favouring OCD in several pairwise comparisons. Housekeeping controls, by contrast, showed little differentiation between the traits, which is what we would expect from a general polygenic background rather than anything trait-specific.

Full standard GSEA and differential GSEA outputs for the 39 evaluated gene sets are provided in Appendix C.

Cross-tissue TWAS: Allergic risk projected onto the brain

Using S-PrediXcan with GTEx version 8 MASHR models, we projected the genetic risk captured by the allergic-disease GWAS onto six brain tissues that matter for OCD circuitry: frontal cortex (Brodmann area 9), hippocampus, amygdala, anterior cingulate cortex (Brodmann area 24), nucleus accumbens, and caudate.

Pruning-related genes turned up repeatedly among the significant results (Table 3). TAP2 produced the strongest single signal (p = 3.02 × 10⁻¹¹ in amygdala), and nine other pruning genes, including ATG5, PSMB8, HLA-B, NFKB1, ROCK2, RELA, HLA-A, EFNA1, and SEMA6A, cleared an FDR threshold of 0.05. Complement genes C4A and C4B showed supportive signals but did not clear FDR in the brain TWAS table, so they are not counted among the FDR-significant brain TWAS pruning genes. The REACTOME Circadian Clock set showed significant overall enrichment as well: its member genes had a mean absolute Z-score 1.12 times higher than the genome-wide background (Mann-Whitney p = 0.019), and six individual genes within the set reached FDR significance. The glutamatergic set was similarly enriched (1.07 times the background, p = 0.013), with four FDR-significant genes. The pruning set produced many individual FDR-significant hits, but its overall Mann-Whitney enrichment against the full TWAS background did not reach significance in the brain analysis (enrichment ratio = 1.05, p = 0.277). Housekeeping controls also produced individual FDR hits, which is not surprising given the high statistical power of the allergic-disease GWAS, but they did not show systematic set-level excess.

**Table 3:** Selected FDR-significant genes in cross-tissue TWAS analyses. TWAS = transcriptome-wide association study; FDR = false discovery rate; MHC = major histocompatibility complex; NF-κB = nuclear factor kappa-light-chain-enhancer of activated B cells. Individual gene-tissue associations were considered FDR-significant at q < 0.05. Some genes are included because they were mechanistically central and nominally associated, but their non-FDR status is shown explicitly.

Gene	Category	Allergic risk in the brain (Best tissue, p/q)	OCD risk in peripheral tissues (Best tissue, p/q)	Functional role in the model
TAP2	MHC/Pruning	Amygdala, p = 3.02 × 10⁻¹¹, q < 0.001	-	MHC class I antigen transport and synaptic tagging context
ATG5	Autophagy/Pruning	Hippocampus, q < 0.001	-	Microglial phagocytosis/autophagy
PSMB8	MHC/Pruning	Anterior cingulate cortex, q < 0.001	-	Immunoproteasome/antigen processing
HLA-B	MHC/Pruning	Hippocampus, q = 0.001	-	MHC class I synaptic tagging context
NFKB1	Immune/Pruning	Caudate, q = 0.001	-	Inflammatory signalling
ROCK2	Oligodendroglial/Pruning	Frontal cortex, q = 0.002	-	Cytoskeletal regulation/myelination context
RELA	Immune/Pruning	Amygdala, q = 0.004	-	NF-κB p65 inflammatory signalling
PSMD3	Circadian Clock	Hippocampus, p = 1.31 × 10⁻⁸, q < 0.001	-	Proteasome-linked circadian output
NR1D1	Circadian Clock	Frontal cortex, p = 1.17 × 10⁻⁴, q = 0.010	Spleen, p = 1.54 × 10⁻³, q = 0.137	REV-ERBα; clock-pruning gate candidate
EZH2	Circadian/chromatin regulator	-	Non-sun-exposed skin, p = 9.14 × 10⁻⁶, q = 0.011	Peripheral circadian-related chromatin signal
RORB	Circadian Clock	Frontal cortex, nominal only	Subcutaneous adipose, p = 5.65 × 10⁻⁴, q = 0.089	Clock and oligodendrocyte-related candidate
CLOCK	Core Clock	Frontal cortex, non-significant	Non-sun-exposed skin, p = 3.08 × 10⁻³, q = 0.194	Core transcription-translation feedback-loop oscillator

Cross-tissue TWAS: OCD risk projected onto skin and peripheral tissues

When we reversed the direction - projecting OCD genetic risk onto skin and allergy-relevant peripheral tissues (sun-exposed and non-sun-exposed skin, lung, whole blood, subcutaneous adipose, liver, and spleen) - the circadian clock sets stood out sharply. The KEGG Circadian Rhythm Mammal set showed the strongest enrichment of any analysis we conducted, at 1.95 times the background level (p = 1.62 × 10⁻⁸). GOBP Regulation of Circadian Rhythm followed closely (1.23 times, p = 6.33 × 10⁻⁸), as did REACTOME Circadian Clock (1.20 times, p = 1.79 × 10⁻⁵). At the individual-gene level, EZH2 in non-sun-exposed skin reached FDR significance within the circadian-related target lists (Z = 4.44, p = 9.14 × 10⁻⁶, q = 0.011). Core clock genes such as NR1D1, RORB, CLOCK, NPAS2, RORA, BTRC, and BMAL1 showed nominal signals in peripheral tissues, but most did not individually clear FDR. The pruning and glutamatergic sets were modestly elevated in these peripheral tissues at the nominal level, with notable individual FDR-significant pruning signals for LYNX1 and MAP1LC3A in spleen and adipose tissue.

Summary of convergence

Taken together, the three layers of analysis - MAGMA gene-set enrichment, standard and differential GSEA, and cross-tissue TWAS - paint a consistent picture (Table 4). Synaptic pruning and circadian gating pathways are enriched in both allergic diseases and OCD, and the gene-level signals go well beyond what broad polygenic overlap alone would predict. The cross-tissue findings are especially telling: genetic variants associated with allergic diseases influence pruning and clock gene expression in brain regions central to OCD, while genetic variants associated with OCD perturb clock gene expression in skin and other peripheral tissues relevant to allergic disease.

**Table 4:** Summary of principal cross-tissue TWAS findings. TWAS = transcriptome-wide association study; OCD = obsessive-compulsive disorder; FDR = false discovery rate; MHC = major histocompatibility complex; NF-κB = nuclear factor kappa-light-chain-enhancer of activated B cells; MAGMA = Multi-marker Analysis of GenoMic Annotation.

Analysis layer	Main finding	Interpretation
Allergic risk projected onto OCD-relevant brain tissues	TAP2 in the amygdala was the strongest target-gene association (p = 3.02 × 10⁻¹¹), with additional FDR-significant MHC, NF-κB, autophagy, and pruning-related genes	Allergic-disease genetic risk maps onto predicted expression of neuroimmune genes in brain regions relevant to OCD
Allergic risk projected onto the brain: circadian sets	REACTOME Circadian Clock showed 1.12-fold enrichment (Mann-Whitney p = 0.019), driven largely by proteasome-linked clock-output genes	Brain circadian signal appears more proteasome-linked than core CLOCK-BMAL1-PER-CRY driven
Allergic risk projected onto the brain: glutamatergic set	Glutamatergic set showed 1.07-fold enrichment (p = 0.013), with FOSL1, EP300, CALML4, and PRKCB among FDR-significant hits	Suggests activity-dependent transcription and calcium signalling may sit alongside pruning biology
OCD risk projected onto peripheral tissues	KEGG Circadian Rhythm Mammal showed 1.95-fold enrichment (p = 1.62 × 10⁻⁸), with GOBP and REACTOME circadian sets also strongly enriched	OCD genetic risk is associated with predicted expression of circadian genes in skin and other allergy-relevant tissues
Peripheral pruning signals	LYNX1 and MAP1LC3A reached FDR significance in peripheral tissues	Pruning/autophagy signals are present but weaker than the peripheral circadian signal
Negative-control behaviour	MAGMA negative controls were not enriched; TWAS housekeeping hits appeared in high-powered analyses but did not show consistent set-level specificity	Supports specificity in the primary MAGMA layer, while warning against over-reading individual TWAS hits

## Discussion

The findings reported here provide, to our knowledge, the first direct head-to-head evidence that OCD and allergic diseases share a meaningful portion of their common-variant architecture at the level of specific biological pathways. When we ran the identical MAGMA framework on the large allergic-diseases GWAS [5] that we had previously applied to the 2025 OCD meta-analysis [9], two broad biological themes stood out: synaptic pruning and circadian clock regulation. The pruning set clearly survived Bonferroni correction in the allergic GWAS, while the REACTOME circadian set showed supportive MAGMA enrichment and stronger support in the standard GSEA and TWAS layers. The genes carrying much of the signal overlapped in a striking way with those that had driven the pruning and circadian findings in OCD. These results move us well past the modest overall genetic correlations reported in earlier cross-trait work and offer something more concrete - a mechanistic bridge for the epidemiological comorbidity that clinicians and researchers have long noticed but struggled to explain [6-8].

What makes this convergence especially convincing is that it holds up against rigorous controls. Neither the housekeeping set nor the monoamine set showed enrichment in the primary MAGMA analysis, which means the signals we observed are unlikely to be artefacts of general polygenic background or of something quirky in the analytic pipeline. On top of that, the pruning enrichment in allergic disease stayed strong even after we stripped out genes that overlap with glutamatergic lists - the same independence we had seen in OCD [10]. The circadian signal, for its part, came through across multiple standardised definitions. REACTOME defines curated biological reactions and pathways; KEGG defines compact canonical pathway maps; WikiPathways provides community-curated pathway models; and Gene Ontology Biological Process sets classify genes by annotated biological processes [18-21]. The fact that circadian signals appeared across several of these different definitions argues against a single-database artefact, although pathway overlap and pathway-size bias remain important caveats.

The cross-tissue transcriptome-wide association analyses added yet another dimension. Projecting allergic genetic risk onto six brain regions central to OCD circuitry, including frontal cortex, anterior cingulate, caudate, nucleus accumbens, hippocampus, and amygdala, yielded multiple FDR-significant hits among pruning and circadian genes. Going the other direction, projecting OCD risk onto skin and other allergy-relevant tissues (sun-exposed and non-sun-exposed skin, lung, blood, adipose, liver, spleen) revealed especially strong enrichment for the circadian clock sets, most notably the KEGG core clock set at 1.95-fold enrichment (p = 1.62 × 10⁻⁸). These are predicted-expression associations, not measured tissue expression in patients. Still, the bidirectional pattern is difficult to ignore and is compatible with a skin-brain axis that may help sustain comorbidity over time.

These findings also fit with, but extend beyond, previous work. Epidemiological studies have already shown OCD-atopy comorbidity at the clinical level [6-8]. OCD neurobiology studies have repeatedly implicated cortico-striatal and cortico-striato-thalamo-cortical dysfunction [2,3]. Separately, developmental neuroscience has shown that complement and microglia can regulate synapse elimination [11], while allergy genetics has shown that asthma, hay fever, and eczema share immune-regulatory risk loci [5]. What had been missing was a direct comparison of pruning and circadian gene sets across OCD and allergic-disease GWAS data using the same pathway framework.

Interpretation of novel genetic convergence

Pulling together the MAGMA, GSEA/differential GSEA (DGSEA), and cross-tissue TWAS results, a coherent picture emerges: common-variant risk for allergic disease and OCD converges on the glial-immune machinery responsible for refining cortico-striato-thalamo-cortical circuits during adolescence. This convergence is not spread thinly across the genome. It is fairly specific. The pruning set's leading genes in allergic disease (HLA-B, TAP2, C4A, NFKB1, SMAD3) are the very same kinds of loci that carried the strongest signals in OCD once we had decomposed the original curated list into standardised oligodendrocyte and MHC markers [10]. The circadian enrichment, driven by PSMD3, NR1D1, and RORB, aligns with the temporal gating layer identified in the subsequent circadian-gated pruning analysis [10]. The MAGMA circadian result in allergic disease should be described cautiously because its Bonferroni-adjusted p-value was 0.015, above the eight-set threshold of 0.0063. Its relevance comes from convergence across GSEA, TWAS, OCD comparator findings, and published skin-clock biology rather than from the MAGMA result alone.

Crucially, the signals are not confined to a single tissue. Allergic variants shape predicted pruning and clock gene expression in brain regions that are critical for habit formation and error monitoring, while OCD variants shape predicted clock expression in skin and lung, precisely the tissues where allergic inflammation originates. This tissue crosstalk goes a long way towards explaining why epidemiological associations persist even though overall SNP-level genetic correlations tend to hover near zero or sometimes go negative with certain inflammatory traits. The genetic architecture seems to be modular: a shared core vulnerability in pruning and its timing, which gets amplified by peripheral immune activation in people who happen to carry the right combination of risk variants.

There are, of course, alternative biological explanations. The MHC signal may partly reflect general immune architecture or long-range linkage disequilibrium rather than synaptic tagging specifically. The circadian signal may partly reflect sleep disruption, itch-related arousal, medication exposure, or stress biology rather than a primary molecular clock disturbance. Healthcare contact bias could also inflate clinical comorbidity estimates, because patients with chronic skin disease may have more opportunities to receive psychiatric diagnoses. Finally, repetitive washing and scratching can worsen barrier dysfunction without requiring a shared genetic mechanism. These explanations are not mutually exclusive with the model proposed here, but they are important safeguards against over-interpreting pathway convergence as causation.

Proposed mechanistic model

**Figure 1:**
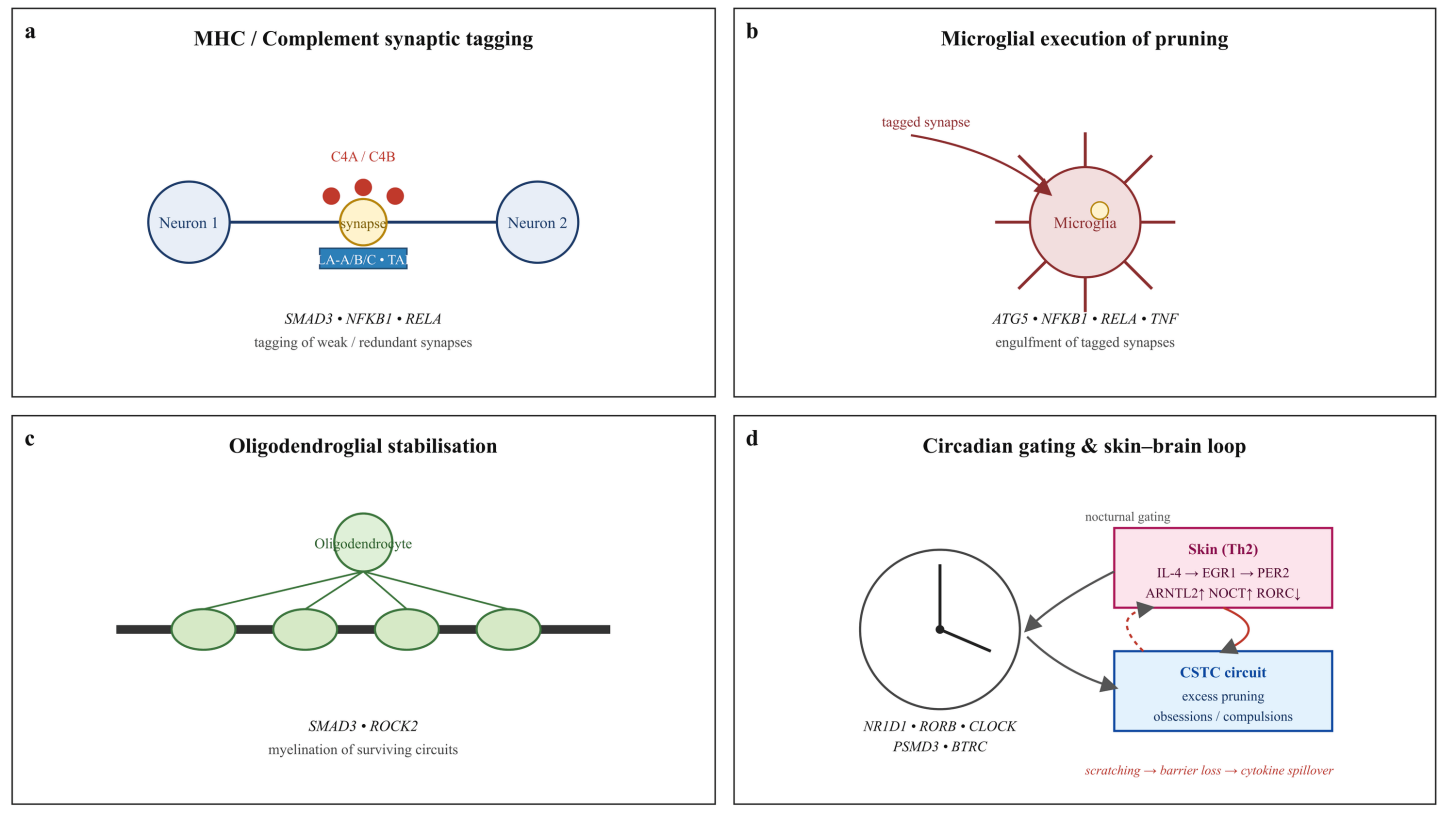
Schematic of the integrated mechanistic model. Genetic risk converges on four interlocking modules: (a) major histocompatibility complex (MHC) and complement tagging of weak or redundant synapses, driven by HLA-A/B/C, TAP2, C4A/C4B, SMAD3, NFKB1 and RELA; (b) microglial execution of pruning through phagocytic and autophagic machinery (ATG5, NFKB1, RELA, TNF); (c) oligodendroglial stabilisation of surviving circuits via SMAD3- and ROCK2-mediated myelination; and (d) circadian gating of these processes by core clock genes (NR1D1, RORB, CLOCK, PSMD3, BTRC). In the proposed model, these modules together increase vulnerability to excessive cortico-striato-thalamo-cortical (CSTC) pruning. Peripheral T helper 2 inflammation in skin, involving IL-4/EGR1/PER2 signalling, ARNTL2 and NOCT upregulation, RORC downregulation, AQP3 dysregulation, and mast-cell FcεRI gating, may amplify the central process through nocturnal itch, scratching, barrier disruption, cytokine spillover and behavioural feedback, forming a self-reinforcing skin–brain neuroimmune loop. Credits: Ngo Cheung. Figure created using Microsoft PowerPoint (Microsoft Corporation, Redmond, WA).

Building on all of these observations, we propose a unified, gene-level model that ties together the findings into what we call a skin-brain neuroimmune loop. The model extends the glial-immune circuit maturation hypothesis [10] and its circadian-gated extension, while folding in published skin clock dysregulation data from atopic dermatitis [25,26]. We should stress that this model is hypothetical and will require functional validation, but the genetic evidence assembled here makes it a reasonable working framework. As shown in Figure 1, risk variants converge on four interlocking modules whose combined effect is excessive synaptic pruning within cortico-striato-thalamo-cortical circuits, amplified by peripheral allergic inflammation and reinforced by behavioural feedback.

The first module centres on MHC and complement tagging of synapses. Both traits show strong enrichment for HLA-B, HLA-A, HLA-C, TAP2, and C4A/C4B. These molecules are responsible for marking weak or redundant synapses so that they can be eliminated [10,11]. In allergic disease specifically, Th2 cytokines, driven by signals at the IL13, IL33, and IL1R1 loci, upregulate local MHC and complement expression on keratinocytes and dendritic cells, which increases allergen penetration and allows systemic spillover. This peripheral signal can then reach the brain and further tag cortico-striato-thalamo-cortical synapses during the adolescent pruning window, when circuits are most vulnerable to over-elimination.

The second module involves microglial execution of the pruning itself. Once synapses have been tagged, microglia engulf them. The key genes here, including NFKB1, RELA, TNF, and ATG5, all showed top signals in the allergic pruning set and multiple FDR-significant hits when allergic risk was projected onto brain tissue through the TWAS. NFKB1 and RELA drive the transcriptional activation of complement and phagocytic programs, while ATG5 is essential for autophagosome formation during the engulfment process. In atopic skin, the accumulation of dendritic cells, which has been shown to correlate inversely with overall clock strength, activates these same pathways and releases cytokines that could prime brain microglia toward excessive pruning.

The third module concerns oligodendroglial stabilisation. Synapses that survive the pruning process still need adequate myelination if the circuit is going to function in a stable way. SMAD3, which was one of the strongest pruning-relevant genes in the allergic MAGMA analysis, and ROCK2, which reached FDR significance in the brain TWAS, both converge on oligodendrocyte differentiation and myelin sheath formation. These two genes were also among the dominant carriers of the OCD pruning signal once we decomposed the curated set into adult oligodendrocyte markers [10]. When myelination falls short, cortico-striato-thalamo-cortical loops may be left hyperexcitable and rigid, which is one plausible circuit phenotype underlying compulsive behaviour.

The fourth module is the circadian gating of pruning windows. None of this pruning machinery runs around the clock; it is gated by the body's circadian system. The circadian signals showing up across the analyses involve genes like PSMD3, NR1D1 (better known as REV-ERB alpha), RORB, CLOCK, and BTRC. Together, these set the daily windows when immune activity, proteasome function, MHC/complement tagging, and microglial responses may be most active. In healthy skin, CLOCK directly controls AQP3 to handle nocturnal hydration and barrier repair, and BMAL1 keeps tight junctions intact. But in atopic dermatitis, clock gene expression drops across the board, with some telling exceptions. ARNTL2 and NOCT get pushed upward, while RORC goes in the opposite direction. What happens next is a kind of self-reinforcing loop: IL-4 triggers EGR1, which ramps up PER2 in keratinocytes, locking in a pro-inflammatory cycle. Meanwhile, mast cells lose the rhythmic check on FcεRI-mediated degranulation, so histamine and IL-31 pour out at night, and that's what drives the intense nocturnal itch and scratching that patients know all too well [25,26].

The model closes with what we see as a self-reinforcing skin-brain feedback loop. Genetic pruning vulnerability, carried by variants in HLA-B, C4A, TAP2, NFKB1, and SMAD3, combines with Th2 cytokine signalling from IL13, IL33, and IL1R1 loci and with circadian misalignment marked by upregulated ARNTL2 and NOCT alongside downregulated RORC, NR1D1, and RORB. Together, these may produce excessive cortico-striato-thalamo-cortical synapse loss, which could contribute to OCD symptoms, i.e., obsessions and compulsions. The compulsions, particularly washing behaviours, then damage the skin barrier further, worsening allergen penetration. More allergen penetration means more Th2 cytokines, which may feed back into further central pruning. This loop offers a natural explanation for why comorbidity tends to be stronger in early-onset and treatment-resistant cases, and why nocturnal symptoms may matter in both conditions.

Again, this is a probable hypothetical model developed from convergent secondary analyses and published literature. It was not directly tested in cells, animals, or longitudinal patient cohorts in the present study.

Clinical and therapeutic implications

This model carries some fairly immediate practical implications, but they should be read as hypothesis-generating rather than as clinical recommendations. To start with, it supports the case for routine screening for allergic disease in OCD patients, especially those whose symptoms appeared early or who have not responded well to first-line treatments, and for screening in the other direction as well. Clinicians managing atopic dermatitis or other allergic conditions in young people might benefit from keeping an eye out for emerging obsessive-compulsive symptoms, particularly when the allergic burden is severe.

Second, the prominence of circadian pathways in both conditions points to chronotherapy as a potentially useful add-on strategy. Evening application of barrier creams, timed melatonin supplementation, or morning bright-light exposure could, in principle, help restore skin clock function and reduce the nocturnal itch that drives so much of the scratching and sleep disruption in atopic patients. If the model we have outlined is on the right track, dampening that nocturnal immune activation might also take some pressure off the central pruning process and, by extension, reduce compulsive behaviours. This possibility has not been tested in the present study and should be evaluated in controlled clinical trials before being translated into practice.

Third, and perhaps most ambitiously, directly targeting the shared glial-immune axis could yield benefits on both fronts simultaneously. Minocycline, which dampens microglial activation and has already shown some promise as an augmentation strategy in OCD [27], might work synergistically with dupilumab, a biologic that blocks IL-4 and IL-13 signalling and is now used for moderate-to-severe atopic dermatitis [28]. Combining the two in comorbid patients, addressing the central pruning arm and the peripheral inflammatory arm at the same time, is something that warrants formal testing in controlled trials. No drug-combination inference should be taken from these genetic analyses alone.

There is also a subtyping angle worth considering. Patients who carry high polygenic risk at loci like HLA-B, C4A, NR1D1, and RORB and who also present with clinical atopy may represent something like an "immune-circadian OCD" endophenotype. If so, these individuals might respond better to immunomodulatory or chronotherapeutic strategies than to standard selective serotonin reuptake inhibitor (SSRI) monotherapy alone. Identifying them early could meaningfully change their treatment trajectory.

Broader neurodevelopmental context

The relevance of this model may extend well beyond OCD and allergy. Excessive complement-mediated pruning has already been implicated in schizophrenia [10,11], and circadian disruption is a shared feature across autism, ADHD, and mood disorders. The skin-brain loop we describe here could represent a more general mechanism through which peripheral immune activation shapes central circuit refinement during development. That would help make sense of the high rates of atopic disease consistently reported in neurodevelopmental cohorts. Future work that traces these pathways across diagnostic boundaries could sharpen transdiagnostic models of psychopathology and, ideally, open up intervention strategies that benefit more than one condition at a time.

Strengths

Several strengths are worth noting. First, the allergic-disease GWAS was large, with more than 360,000 participants, giving the pathway analyses adequate power. Second, the same MAGMA framework was used across allergic diseases and OCD, reducing analytic asymmetry. Third, the study included negative-control gene sets, which helped separate pruning and circadian signals from general polygenic or housekeeping background. Fourth, the analysis did not stop at one method: MAGMA, standard GSEA, differential GSEA, and cross-tissue TWAS all pointed toward overlapping immune-circadian biology, even though each method asks a slightly different question. Finally, the bidirectional TWAS design made the model more biologically interpretable by asking whether allergic risk appears in brain-relevant tissues and whether OCD risk appears in allergy-relevant peripheral tissues.

Limitations and future directions

A number of limitations need to be acknowledged. Both GWAS datasets are drawn predominantly from European-ancestry samples, which limits how far we can generalise the findings. Replication in more diverse populations is essential before the pathway convergence described here can be considered universal. Although both datasets were broadly European, they were not identical in cohort composition, ascertainment, or ancestry substructure. The use of a 1000 Genomes European linkage disequilibrium panel helped keep the analysis internally consistent, but it cannot fully remove ancestry mismatch.

A second limitation is that the study compared pathway enrichment results across two independent GWAS datasets rather than performing a formal cross-trait genetic analysis. We did not conduct cross-trait linkage disequilibrium score regression, colocalisation, genomic structural equation modelling, or Mendelian randomisation between OCD and allergic diseases in this study. Therefore, the results cannot conclusively demonstrate shared causal genetic architecture. They show convergent pathway signals.

A third limitation is possible confirmation bias. The eight primary gene sets were preselected from the author's earlier OCD-focused study, which helped preserve hypothesis-driven comparability but could also favour rediscovery of the same biological themes. The inclusion of public standardised circadian sets, glial marker sets, and negative controls partly reduces this concern, but independent replication using externally curated gene sets would still be important.

A fourth limitation is that enrichment analyses are inherently exploratory and can be affected by pathway size, gene length, linkage disequilibrium structure, gene density, and gene-set overlap. The KEGG core clock set is very small, while the WikiPathways and Gene Ontology circadian sets are much broader; these differences can change both power and interpretation. The MHC region also has complex linkage disequilibrium, meaning that gene-level signals in that region should be interpreted carefully.

While the pathway-level overlap is robust, we cannot claim causality on the basis of these analyses alone. Mendelian randomisation studies and longitudinal cohorts that track synaptic density, myelin integrity, skin barrier function, and symptom onset in high-risk adolescents will be needed to establish directionality. The model we have proposed remains hypothetical, and functional validation is the obvious next step. Cell-type-specific CRISPR (clustered regularly interspaced short palindromic repeats) editing of convergent genes (HLA-B, NR1D1, and SMAD3, for instance) in human iPSC-derived microglia, oligodendrocytes, and keratinocytes, followed by co-culture experiments, would allow researchers to test the directional effects we have inferred from statistical overlap.

The present study also did not generate new skin transcriptomic data. The atopic-dermatitis clock-gene findings were taken from published transcriptomic and bioinformatic studies and used to contextualise the genetic results [25,26]. Similarly, S-PrediXcan estimates genetically regulated expression; it does not measure real-time gene expression in patients with OCD, allergic disease, or both.

Looking ahead, several lines of work seem especially promising. Composite polygenic risk scores that integrate pruning and circadian variants could be developed to predict comorbidity risk before symptoms emerge. Multimodal imaging studies combining synaptic PET, myelin water imaging, and skin clock profiling could test the model's predictions at the level of individual patients. Proof-of-concept trials combining chronotherapy and immunomodulation in comorbid patients would put the therapeutic implications to a direct test. As larger multi-ancestry GWAS become available and single-cell expression quantitative trait locus (eQTL) resources grow in depth and coverage, it will become possible to refine the tissue-specific and cell-type-specific contributions with much greater precision than we can manage today.

## Conclusions

The present study shows that OCD and allergic diseases share a convergent polygenic pattern that centres on synaptic pruning and circadian gating. By bringing together genome-wide pathway analyses, cross-tissue transcriptome-wide association studies, and published atopic-dermatitis clock-gene expression evidence, we have arrived at a detailed gene-level mechanistic model, one in which MHC/complement tagging, microglial execution, oligodendroglial stabilisation, and circadian regulation interact with peripheral Th2 inflammation to drive a probable and hypothetical self-reinforcing skin-brain loop. This framework helps account for the observed comorbidity between the two conditions and identifies pathways that could be tested in future mechanistic and treatment studies. It should not be read as causal proof or as direct evidence for any specific clinical intervention. We would encourage the field to move past single-disorder silos and begin testing integrated immune-circadian hypotheses in patients who carry both diagnoses. If such approaches prove effective, they could meaningfully change outcomes for the millions of people worldwide who live with OCD and allergic disease together.

## Appendices

Appendix A: OCD GWAS → TWAS in atopic-related peripheral tissues

S-PrediXcan Multi-Gene-Set Transcriptome-Wide Association Analysis (GTEx v8 MASHR Models)

GWAS: OCD 2025 (Nca/Nco configuration; Neff = 68,099; 6,791,632 SNPs). Tissues: skin (not-sun-exposed, sun-exposed), lung, whole blood, subcutaneous adipose, liver, and spleen. Models: Zenodo DOI 10.5281/zenodo.3518299.

**Table 5:** Gene-set-level TWAS enrichment summary across seven peripheral tissues. Note. Sorted by enrichment p-value. Enrichment ratio = mean |Z| for target genes divided by mean |Z| for all other TWAS genes (reference mean |Z| = 0.927). Mann-Whitney p tests whether target-gene |Z| exceeds background. Mean enrichment ratio for circadian target sets = 1.25× vs. controls = 1.01×. *** P < 0.001. TWAS = transcriptome-wide association study; FDR = false discovery rate.

Gene set	n, Target	n, Tested	Nominal (p < 0.05)	FDR (q < 0.05)	Enrichment ratio	Mann–Whitney p
KEGG_Circadian_Rhythm_Mammal	13	10	4	0	1.95×	1.62 × 10⁻⁸***
GOBP_Regulation_of_Circadian_Rhythm	107	79	23	1	1.23×	6.33 × 10⁻⁸***
Reactome_Circadian_Clock	104	88	22	0	1.20×	1.79 × 10⁻⁵***
Negative_Controls_Housekeeping	182	147	29	2	1.06×	.229
Glutamatergics	130	111	23	0	1.05×	.052
Pruning	213	184	32	2	1.03×	.163
WP_Circadian_Rhythm_Genes	171	127	23	1	1.01×	.428
Monoamines	101	78	12	1	0.96×	.989

**Table 6:** S-PrediXcan performance and significance per tissue. Note. Bonferroni threshold α computed per tissue as 0.05 ÷ genes tested. FDR = false discovery rate.

Tissue	Model genes	Genes tested	Skipped	Nominal (p < 0.05)	Bonferroni Sig.
Skin, Not-Sun-Exposed (Suprapubic)	14,932	12,060	2,872	1,116	6 (α = 4.15 × 10⁻⁶)
Skin, Sun-Exposed (Lower Leg)	15,204	12,325	2,879	1,182	6 (α = 4.06 × 10⁻⁶)
Lung	15,058	12,037	3,021	1,105	7 (α = 4.15 × 10⁻⁶)
Whole Blood	12,623	10,111	2,512	916	12 (α = 4.95 × 10⁻⁶)
Adipose, Subcutaneous	14,732	11,863	2,869	1,107	7 (α = 4.21 × 10⁻⁶)
Liver	12,714	9,707	3,007	831	3 (α = 5.15 × 10⁻⁶)
Spleen	14,073	10,989	3,084	1,038	7 (α = 4.55 × 10⁻⁶)
Total gene-tissue associations	79,092 associations across 17,772 unique genes; 221 FDR-significant (117 unique genes); 41 Bonferroni-significant (26 unique genes)				

**Table 7:** Top TWAS associations in circadian gene sets (best tissue per gene). Note. Circadian-related target genes with nominal p < 0.05 are shown at their best (lowest-p) tissue. KEGG core clock genes (CRY1/2, PER2/3, BHLHE41) included for completeness. ✓ indicates membership; — indicates absence. TWAS = transcriptome-wide association study; FDR = false discovery rate; Reactome = Reactome_Circadian_Clock; KEGG = KEGG_Circadian_Rhythm_Mammal; WP = WikiPathways Circadian Rhythm Genes (WP3594); GOBP = GOBP_Regulation_of_Circadian_Rhythm. * FDR q < 0.05.

Gene	Best tissue	Z	p	FDR (q)	Sig.	Reactome	KEGG	WP	GOBP
EZH2	Skin, Not-Sun-Exp.	4.437	9.14 × 10⁻⁶	.011	*	—	—	✓	✓
TBL1XR1	Skin, Not-Sun-Exp.	−3.689	2.25 × 10⁻⁴	.064	—	✓	—	—	—
SPSB4	Whole Blood	3.559	3.72 × 10⁻⁴	.074	—	—	—	—	✓
CSNK2B	Whole Blood	−3.510	4.48 × 10⁻⁴	.081	—	✓	—	—	—
RORB	Adipose, Subcut.	3.448	5.65 × 10⁻⁴	.089	—	✓	—	✓	✓
PSMD6	Liver	−3.397	6.81 × 10⁻⁴	.096	—	✓	—	—	—
NR1D1	Spleen	3.168	1.54 × 10⁻³	.137	—	✓	✓	✓	✓
PSPC1	Skin, Sun-Exp.	−3.116	1.83 × 10⁻³	.148	—	—	—	✓	✓
RORA	Liver	−3.095	1.97 × 10⁻³	.155	—	✓	—	✓	✓
BTRC	Spleen	3.037	2.39 × 10⁻³	.170	—	✓	—	✓	✓
CLOCK	Skin, Not-Sun-Exp.	2.959	3.08 × 10⁻³	.194	—	✓	✓	✓	✓
NPAS2	Skin, Not-Sun-Exp.	2.899	3.74 × 10⁻³	.214	—	✓	✓	—	—
PPP1CB	Liver	2.847	4.41 × 10⁻³	.232	—	✓	—	✓	✓
MAPK8	Spleen	2.686	7.23 × 10⁻³	.288	—	—	—	✓	✓
SEM1	Lung	−2.719	6.55 × 10⁻³	.275	—	✓	—	—	—
CSNK2A2	Skin, Not-Sun-Exp.	−2.636	8.40 × 10⁻³	.303	—	✓	—	—	—
USP7	Lung	2.528	1.15 × 10⁻²	.344	—	—	—	—	✓
PSMC5	Skin, Not-Sun-Exp.	2.473	1.34 × 10⁻²	.358	—	✓	—	—	—
ADIPOQ	Adipose, Subcut.	2.448	1.44 × 10⁻²	.366	—	—	—	✓	—
NMS	Skin, Not-Sun-Exp.	−2.454	1.41 × 10⁻²	.365	—	—	—	✓	—
PSMB4	Skin, Not-Sun-Exp.	2.407	1.61 × 10⁻²	.380	—	✓	—	—	—
MAGEL2	Skin, Sun-Exp.	−2.369	1.78 × 10⁻²	.392	—	—	—	✓	✓
PSMD2	Whole Blood	2.363	1.81 × 10⁻²	.395	—	✓	—	—	—
GHRL	Liver	2.264	2.35 × 10⁻²	.434	—	—	—	✓	✓
SIAH2	Skin, Not-Sun-Exp.	2.266	2.35 × 10⁻²	.434	—	—	—	—	✓
CUL1	Lung	−2.290	2.20 × 10⁻²	.427	—	✓	—	✓	✓
PSMD14	Skin, Sun-Exp.	−2.257	2.40 × 10⁻²	.437	—	✓	—	—	—
NGFR	Lung	−2.234	2.55 × 10⁻²	.448	—	—	—	✓	—
HDAC1	Skin, Not-Sun-Exp.	−2.215	2.68 × 10⁻²	.455	—	—	—	✓	—
PARP1	Whole Blood	2.194	2.82 × 10⁻²	.461	—	—	—	—	✓
BMAL1 (ARNTL)	Lung	2.180	2.93 × 10⁻²	.467	—	✓	✓	✓	✓
PSMC3	Whole Blood	−2.158	3.10 × 10⁻²	.477	—	✓	—	—	—
FAS	Lung	−2.137	3.26 × 10⁻²	.483	—	—	—	✓	—
FBXW7	Skin, Sun-Exp.	2.128	3.34 × 10⁻²	.485	—	—	—	—	✓
FBXL3	Spleen	−2.103	3.55 × 10⁻²	.493	—	✓	—	✓	✓
BHLHE41	Skin, Not-Sun-Exp.	−1.934	5.31 × 10⁻²	.550	—	—	✓	—	—
CRY2	Whole Blood	1.820	6.88 × 10⁻²	.590	—	—	✓	—	—
PER3	Skin, Sun-Exp.	1.111	2.67 × 10⁻¹	.796	—	—	✓	—	—
PER2	Whole Blood	1.106	2.69 × 10⁻¹	.797	—	—	✓	—	—
CRY1	Whole Blood	−1.038	2.99 × 10⁻¹	.812	—	—	✓	—	—

**Table 8:** Top TWAS associations in non-circadian gene sets (best tissue per gene). Note. All genes with nominal p < 0.05 from Housekeeping, Glutamatergic, Pruning, and Monoamine target sets at their best (lowest-p) tissue. TWAS = transcriptome-wide association study; FDR = false discovery rate; DA = dopamine; NE = norepinephrine; Mito Cplx = mitochondrial complex; TCA = tricarboxylic-acid cycle. * FDR q < 0.05. ** FDR q < 0.01.

Gene	Best tissue	Z	p	FDR (q)	Sig.	Gene set
NDUFA2	Adipose, Subcutaneous	5.297	1.17 × 10⁻⁷	.002	**	Housekeeping (Mito Cplx I)
LYNX1	Spleen	−4.544	5.52 × 10⁻⁶	.008	**	Pruning
RPS5	Skin, Sun-Exposed	−4.893	9.93 × 10⁻⁷	.003	**	Housekeeping (Ribosomal)
MAP1LC3A	Adipose, Subcutaneous	4.166	3.10 × 10⁻⁵	.023	*	Pruning
VAMP2	Liver	4.082	4.47 × 10⁻⁵	.030	*	Monoamines
BCL2L1	Lung	−3.704	2.12 × 10⁻⁴	.062	—	Pruning
C4A	Lung	3.635	2.78 × 10⁻⁴	.068	—	Pruning
HSPA1A	Skin, Sun-Exposed	3.596	3.23 × 10⁻⁴	.069	—	Housekeeping (Heat Shock)
HSPA1L	Skin, Not-Sun-Exp.	−3.596	3.23 × 10⁻⁴	.069	—	Housekeeping (Heat Shock)
RPL4	Skin, Sun-Exposed	−3.567	3.61 × 10⁻⁴	.073	—	Housekeeping (Ribosomal)
ACO2	Lung	3.551	3.83 × 10⁻⁴	.075	—	Housekeeping (TCA)
GLS2	Whole Blood	−3.482	4.97 × 10⁻⁴	.085	—	Glutamatergics
EP300	Lung	3.464	5.32 × 10⁻⁴	.086	—	Glutamatergics
PRKAR2A	Skin, Not-Sun-Exp.	−3.410	6.50 × 10⁻⁴	.095	—	Glutamatergics
C4B	Lung	−3.386	7.10 × 10⁻⁴	.098	—	Pruning
SEMA3F	Lung	3.360	7.79 × 10⁻⁴	.102	—	Pruning
RPS11	Lung	−3.345	8.24 × 10⁻⁴	.105	—	Housekeeping (Ribosomal)
HTR1B	Skin, Sun-Exposed	3.229	1.24 × 10⁻³	.126	—	Monoamines
RPL13	Skin, Not-Sun-Exp.	−3.194	1.40 × 10⁻³	.131	—	Housekeeping (Ribosomal)
CAMK2D	Spleen	−3.171	1.52 × 10⁻³	.137	—	Glutamatergics
PRKACB	Adipose, Subcutaneous	−3.149	1.64 × 10⁻³	.141	—	Glutamatergics
MDH2	Whole Blood	2.990	2.79 × 10⁻³	.185	—	Housekeeping (TCA)
GRIP1	Skin, Sun-Exposed	2.945	3.23 × 10⁻³	.200	—	Glutamatergics
DLGAP1	Adipose, Subcutaneous	2.934	3.35 × 10⁻³	.204	—	Glutamatergics
HDC	Adipose, Subcutaneous	2.929	3.40 × 10⁻³	.204	—	Monoamines (Histamine)
STX1B	Spleen	−2.894	3.81 × 10⁻³	.216	—	Monoamines
PLXNB2	Skin, Not-Sun-Exp.	−2.875	4.04 × 10⁻³	.223	—	Pruning
GAD1	Adipose, Subcutaneous	−2.822	4.77 × 10⁻³	.239	—	Glutamatergics
HTR6	Spleen	−2.775	5.52 × 10⁻³	.255	—	Monoamines
HNMT	Lung	2.755	5.88 × 10⁻³	.263	—	Monoamines (Histamine)
GNB1	Whole Blood	2.743	6.08 × 10⁻³	.267	—	Monoamines (Downstream)
EFNA5	Spleen	2.727	6.39 × 10⁻³	.273	—	Pruning
SPARCL1	Adipose, Subcutaneous	−2.683	7.29 × 10⁻³	.290	—	Pruning
HSPA4	Skin, Not-Sun-Exp.	2.667	7.66 × 10⁻³	.296	—	Housekeeping (Heat Shock)
GFAP	Skin, Not-Sun-Exp.	−2.652	8.00 × 10⁻³	.298	—	Pruning
FBXO45	Skin, Not-Sun-Exp.	2.642	8.24 × 10⁻³	.302	—	Pruning
ENO1	Skin, Not-Sun-Exp.	2.628	8.59 × 10⁻³	.306	—	Housekeeping (Glycolysis)
CAMK2G	Adipose, Subcutaneous	2.590	9.59 × 10⁻³	.321	—	Glutamatergics
SLC1A3	Skin, Not-Sun-Exp.	−2.577	9.97 × 10⁻³	.325	—	Glutamatergics
FH	Whole Blood	−2.573	1.01 × 10⁻²	.327	—	Housekeeping (TCA)
GRIK4	Adipose, Subcutaneous	−2.567	1.03 × 10⁻²	.329	—	Glutamatergics
DFNA5	Whole Blood	2.538	1.12 × 10⁻²	.340	—	Pruning
BAK1	Lung	−2.510	1.21 × 10⁻²	.350	—	Pruning
COX7A2L	Skin, Sun-Exposed	−2.496	1.26 × 10⁻²	.352	—	Housekeeping (Mito Cplx IV)
COX8A	Skin, Sun-Exposed	2.428	1.52 × 10⁻²	.373	—	Housekeeping (Mito Cplx IV)
PRKAR1B	Liver	2.426	1.53 × 10⁻²	.375	—	Glutamatergics
THBS4	Liver	2.423	1.54 × 10⁻²	.376	—	Pruning
GNB5	Skin, Not-Sun-Exp.	2.383	1.72 × 10⁻²	.388	—	Monoamines (Downstream)
GNB3	Skin, Sun-Exposed	−2.380	1.73 × 10⁻²	.389	—	Monoamines (Downstream)
QDPR	Lung	2.351	1.87 × 10⁻²	.398	—	Monoamines
COX5A	Spleen	−2.358	1.84 × 10⁻²	.397	—	Housekeeping (Mito Cplx IV)
NDUFA3	Spleen	−2.346	1.90 × 10⁻²	.401	—	Housekeeping (Mito Cplx I)
PCDHGC3	Whole Blood	2.339	1.93 × 10⁻²	.403	—	Pruning
WNT5A	Liver	−2.334	1.96 × 10⁻²	.407	—	Pruning
GRIK3	Skin, Sun-Exposed	2.333	1.96 × 10⁻²	.406	—	Glutamatergics
TIMP2	Skin, Not-Sun-Exp.	2.328	1.99 × 10⁻²	.406	—	Pruning
ERBB4	Lung	2.315	2.06 × 10⁻²	.413	—	Pruning
CALML6	Liver	2.278	2.27 × 10⁻²	.431	—	Glutamatergics
DBH	Skin, Not-Sun-Exp.	2.269	2.33 × 10⁻²	.434	—	Monoamines (NE)
TNF	Skin, Sun-Exposed	2.244	2.48 × 10⁻²	.443	—	Pruning
CYP2D6	Lung	−2.239	2.52 × 10⁻²	.445	—	Glutamatergics
PRKAR1A	Adipose, Subcutaneous	2.232	2.56 × 10⁻²	.448	—	Glutamatergics
DRD2	Lung	−2.218	2.66 × 10⁻²	.454	—	Monoamines (DA)
HSP90B1	Adipose, Subcutaneous	−2.297	2.16 × 10⁻²	.423	—	Housekeeping (Heat Shock)
DLGAP2	Skin, Not-Sun-Exp.	−2.131	3.31 × 10⁻²	.484	—	Glutamatergics
PRKCA	Lung	−2.113	3.46 × 10⁻²	.490	—	Glutamatergics
JUND	Skin, Not-Sun-Exp.	−2.108	3.51 × 10⁻²	.491	—	Glutamatergics
SLC17A7	Adipose, Subcutaneous	2.073	3.82 × 10⁻²	.506	—	Glutamatergics
TUBB3	Skin, Not-Sun-Exp.	−2.963	3.04 × 10⁻³	.193	—	Housekeeping (Tubulin)
TUBA8	Lung	2.776	5.51 × 10⁻³	.255	—	Housekeeping (Tubulin)
PFN1	Lung	−2.730	6.34 × 10⁻³	.272	—	Housekeeping (Actin)

**Table 9:** Summary of principal findings. Note. Prediction models: GTEx v8 MASHR eQTL (Zenodo DOI: 10.5281/zenodo.3518299). GWAS: OCD 2025 harmonised summary statistics (Neff = 68,099). Results demonstrate reproducible circadian-pathway transcriptomic signal in atopy-related peripheral tissues despite OCD being a neuropsychiatric trait. OCD = obsessive-compulsive disorder; FDR = false discovery rate; WP: WikiPathways; GOBP: Gene Ontology Biological Process.

Domain	Finding
Strongest circadian enrichment	KEGG_Circadian_Rhythm_Mammal showed the largest mean |Z| enrichment (1.95×; Mann-Whitney p = 1.62 × 10⁻⁸), followed by GOBP_Regulation_of_Circadian_Rhythm (1.23×; p = 6.33 × 10⁻⁸) and Reactome_Circadian_Clock (1.20×; p = 1.79 × 10⁻⁵).
Circadian target gene (FDR sig.)	EZH2 (Skin, Not-Sun-Exposed) was FDR-significant in both WP and GOBP circadian sets (Z = 4.44, p = 9.14 × 10⁻⁶, q = 0.011).
Consistent clock-gene pattern	Core clock regulators (NR1D1, RORA, RORB, CLOCK, NPAS2, BMAL1, BTRC, FBXL3) reached nominal significance in peripheral tissues, predominantly the spleen, liver, skin, and adipose.
Target-vs-control contrast	Mean enrichment of circadian target sets (1.25×) exceeded non-circadian controls (1.01×); Monoamines (0.96×) and Housekeeping (1.06×) were non-significant.
Strongest single-gene hit	NDUFA2 in adipose subcutaneous (Z = 5.30, p = 1.17 × 10⁻⁷, q = .002) — housekeeping mitochondrial complex I.
Synaptic-pruning signal	LYNX1 (spleen, q = .008) and MAP1LC3A (adipose, q = .023) were FDR-significant; complement C4A/C4B approached significance in lung (p < 7.1 × 10⁻⁴).
Monoaminergic signal	Only VAMP2 (Liver; q = .030) surpassed FDR; the broader monoamine set did not show excess enrichment (0.96×; Mann-Whitney p = .989).
Global numbers	79,092 gene-tissue tests across 7 tissues and 17,772 unique genes; 221 FDR-significant associations (117 unique genes); 41 Bonferroni-significant (26 unique genes).

Appendix B: Atopic diseases GWAS → TWAS in brain tissues

S-PrediXcan Multi-Gene-Set Transcriptome-Wide Association Analysis (GTEx v8 MASHR Models)

GWAS: Allergic disease meta-analysis, EBI-a-GCST005038 (N = 360,838; 8,117,496 SNPs). Brain tissues: Frontal cortex BA9, hippocampus, amygdala, anterior cingulate cortex BA24, nucleus accumbens, and caudate. Models: Zenodo; DOI: 10.5281/zenodo.3518299.

**Table 10:** Gene-set-level TWAS enrichment summary across six brain tissues. Note. Sorted by enrichment ratio. Enrichment ratio = mean |Z| for target genes ÷ mean |Z| for all other TWAS genes (reference mean |Z| = 0.995). Mean enrichment ratio — target sets = 1.02×; control (housekeeping + pruning) = 1.05×. In contrast to peripheral-tissue results, the circadian signal in the brain is weaker, while Reactome Circadian Clock (proteasome-dense pathway) and Glutamatergics reach nominal enrichment. * P < 0.05. TWAS = transcriptome-wide association study; FDR = false discovery rate.

Gene set	n, Target	n, Tested	Nominal (p < 0.05)	FDR (q < 0.05)	Enrichment ratio	Mann-Whitney p
Reactome_Circadian_Clock	104	87	23	6	1.12×	.019*
Glutamatergics	130	104	21	4	1.07×	.013*
Negative_Controls_Housekeeping	182	140	27	9	1.07×	.220
Pruning	213	178	38	10	1.05×	.277
Monoamines	101	73	15	3	1.02×	.766
GOBP_Regulation_of_Circadian_Rhythm	107	79	16	3	1.00×	.301
WP_Circadian_Rhythm_Genes	171	129	26	5	1.00×	.162
KEGG_Circadian_Rhythm_Mammal	13	9	2	1	0.88×	.916

**Table 11:** S-PrediXcan performance and significance per brain tissue. Note. Bonferroni threshold α computed per tissue as 0.05 ÷ genes tested. The large number of brain TWAS hits reflects the high statistical power afforded by the atopic-disease GWAS (N = 360,838). TWAS = transcriptome-wide association study; GWAS = genome-wide association study; FDR = false discovery rate.

Tissue	Model genes	Genes tested	Skipped	Nominal (p < 0.05)	Bonferroni Sig.
Frontal Cortex (BA9)	14,091	11,861	2,230	1,408	77 (α = 4.22 × 10⁻⁶)
Hippocampus	13,526	11,357	2,169	1,300	57 (α = 4.40 × 10⁻⁶)
Amygdala	12,814	10,605	2,209	1,202	53 (α = 4.71 × 10⁻⁶)
Anterior Cingulate Cortex (BA24)	13,528	11,295	2,233	1,336	56 (α = 4.43 × 10⁻⁶)
Nucleus Accumbens (Basal Ganglia)	14,062	11,835	2,227	1,349	78 (α = 4.22 × 10⁻⁶)
Caudate (Basal Ganglia)	14,118	11,907	2,211	1,407	69 (α = 4.20 × 10⁻⁶)
Total gene–tissue associations	68,860 associations across 16,426 unique genes; 1,359 FDR-significant (535 unique genes); 348 Bonferroni-significant (149 unique genes)				

**Table 12:** FDR-significant TWAS associations across all target gene sets (best tissue per gene). Note. All target-gene associations reaching FDR q < 0.05 at their best (lowest-p) tissue. TWAS = transcriptome-wide association study; FDR = false discovery rate; MHC = major histocompatibility complex; TCA = tricarboxylic-acid cycle; Mito Cplx = mitochondrial complex; DA = dopamine; 5-HT = serotonin; NF-κB = nuclear factor κB; KEGG = Kyoto Encyclopedia of Genes and Genomes; WP = WikiPathways; GOBP = Gene Ontology Biological Process. * q < 0.05. ** q < 0.01. *** q < 0.001.

Gene	Best tissue	Z	p	FDR (q)	Sig.	Gene set
TAP2	Amygdala	6.645	3.02 × 10⁻¹¹	< .001	***	Pruning
ACO2	Nucleus Accumbens	6.423	1.34 × 10⁻¹⁰	< .001	***	Housekeeping (TCA)
PNMT	Frontal Cortex (BA9)	−5.999	1.99 × 10⁻⁹	< .001	***	Monoamines (Epinephrine Synth.)
PSMD3	Hippocampus	−5.684	1.31 × 10⁻⁸	< .001	***	Reactome Circadian Clock
PSMB7	Caudate	−5.507	3.65 × 10⁻⁸	< .001	***	Reactome Circadian Clock
ATG5	Hippocampus	5.472	4.44 × 10⁻⁸	< .001	***	Pruning
HSPA1B	Frontal Cortex (BA9)	5.259	1.45 × 10⁻⁷	< .001	***	Housekeeping (Heat Shock)
PSMB8	Anterior Cing. Cortex	−4.898	9.70 × 10⁻⁷	< .001	***	Pruning/MHC
PPP1R1B	Amygdala	4.859	1.18 × 10⁻⁶	< .001	***	Monoamines (DARPP-32)
RPS16	Nucleus Accumbens	4.791	1.66 × 10⁻⁶	< .001	***	Housekeeping (Ribosomal)
HTR1D	Nucleus Accumbens	−4.700	2.61 × 10⁻⁶	< .001	***	Monoamines (5-HT)
RPS11	Hippocampus	−4.611	4.02 × 10⁻⁶	< .001	***	Housekeeping (Ribosomal)
RPL13A	Frontal Cortex (BA9)	4.561	5.09 × 10⁻⁶	< .001	***	Housekeeping (Ribosomal)
PSMA6	Anterior Cing. Cortex	4.449	8.63 × 10⁻⁶	.001	**	Reactome Circadian Clock
HLA-B	Hippocampus	−4.442	8.90 × 10⁻⁶	.001	**	Pruning/MHC
NFKB1	Caudate	4.441	8.97 × 10⁻⁶	.001	**	Pruning
ROCK2	Frontal Cortex (BA9)	4.377	1.20 × 10⁻⁵	.002	**	Pruning
FOSL1	Amygdala	4.345	1.39 × 10⁻⁵	.002	**	Glutamatergics
EP300	Frontal Cortex (BA9)	4.311	1.62 × 10⁻⁵	.002	**	Glutamatergics
RPL5	Frontal Cortex (BA9)	4.281	1.86 × 10⁻⁵	.002	**	Housekeeping (Ribosomal)
CIART	Anterior Cing. Cortex	4.158	3.21 × 10⁻⁵	.004	**	WP Circadian
RELA	Amygdala	4.117	3.84 × 10⁻⁵	.004	**	Pruning (NF-κB)
HLA-A	Amygdala	−3.951	7.77 × 10⁻⁵	.008	**	Pruning/MHC
NR1D1	Frontal Cortex (BA9)	−3.853	1.17 × 10⁻⁴	.010	*	Reactome/KEGG/WP/GOBP
CALML4	Anterior Cing. Cortex	−3.828	1.29 × 10⁻⁴	.011	*	Glutamatergics
NAGLU	Amygdala	3.800	1.45 × 10⁻⁴	.012	*	WP Circadian
HSPA2	Anterior Cing. Cortex	−3.691	2.24 × 10⁻⁴	.017	*	Housekeeping (Heat Shock)
EFNA1	Nucleus Accumbens	−3.670	2.43 × 10⁻⁴	.018	*	Pruning
FAS	Amygdala	3.646	2.66 × 10⁻⁴	.020	*	WP Circadian
PRKCB	Caudate	−3.641	2.72 × 10⁻⁴	.020	*	Glutamatergics
NDUFA2	Anterior Cing. Cortex	3.514	4.41 × 10⁻⁴	.028	*	Housekeeping (Mito Cplx I)
OPN5	Frontal Cortex (BA9)	3.522	4.28 × 10⁻⁴	.028	*	GOBP Circadian
SEMA6A	Hippocampus	−3.458	5.44 × 10⁻⁴	.033	*	Pruning
BTRC	Frontal Cortex (BA9)	3.448	5.64 × 10⁻⁴	.034	*	Reactome/WP/GOBP
PSMA4	Hippocampus	−3.435	5.92 × 10⁻⁴	.036	*	Reactome Circadian Clock
HSPA1L	Nucleus Accumbens	3.411	6.48 × 10⁻⁴	.038	*	Housekeeping (Heat Shock)

**Table 13:** Top Reactome Circadian-Clock and Core Clock-Gene TWAS associations. Note. The Reactome Circadian Clock signal in the brain is dominated by proteasome subunits (PSMD3, PSMB7, PSMA6, PSMA4), not by canonical clock transcription factors, contrasting with the peripheral-tissue result where CLOCK, NPAS2 and NR1D1 were most prominent. The core KEGG clock oscillator (CLOCK, BMAL1, CRY1/2, PER2) is attenuated in brain TWAS. * q < 0.05. ** q < 0.01. *** q < 0.001. TWAS = transcriptome-wide association study; FDR = false discovery rate; KEGG = Kyoto Encyclopedia of Genes and Genomes.

Gene	Best tissue	Z	p	FDR (q)	Sig.	Category
PSMD3	Hippocampus	−5.684	1.31 × 10⁻⁸	< .001	***	Proteasome
PSMB7	Caudate	−5.507	3.65 × 10⁻⁸	< .001	***	Proteasome
PSMA6	Anterior Cing. Cortex	4.449	8.63 × 10⁻⁶	.001	**	Proteasome
NR1D1 (Rev-ErbA)	Frontal Cortex (BA9)	−3.853	1.17 × 10⁻⁴	.010	*	Core clock
BTRC	Frontal Cortex (BA9)	3.448	5.64 × 10⁻⁴	.034	*	Clock regulator
PSMA4	Hippocampus	−3.435	5.92 × 10⁻⁴	.036	*	Proteasome
PSMB4	Hippocampus	2.867	4.15 × 10⁻³	.126	—	Proteasome
PSMB2	Frontal Cortex (BA9)	−2.837	4.56 × 10⁻³	.132	—	Proteasome
HELZ2	Nucleus Accumbens	−2.829	4.67 × 10⁻³	.134	—	Nuclear receptor coactivator
NCOA1	Caudate	−2.825	4.72 × 10⁻³	.135	—	Nuclear receptor coactivator
RAI1	Caudate	−2.707	6.78 × 10⁻³	.166	—	Clock regulator
PPP1CB	Frontal Cortex (BA9)	−2.276	2.28 × 10⁻²	.309	—	Phosphatase
PSMD11	Frontal Cortex (BA9)	−2.274	2.30 × 10⁻²	.310	—	Proteasome
PSMD8	Frontal Cortex (BA9)	2.192	2.84 × 10⁻²	.340	—	Proteasome
MED1	Nucleus Accumbens	−2.162	3.06 × 10⁻²	.349	—	Mediator complex
PSMB5	Caudate	−2.143	3.21 × 10⁻²	.356	—	Proteasome
RORB	Frontal Cortex (BA9)	−2.100	3.58 × 10⁻²	.373	—	Core clock
TBL1XR1	Hippocampus	−2.077	3.78 × 10⁻²	.382	—	Transcription corepressor
NPAS2	Anterior Cing. Cortex	2.053	4.00 × 10⁻²	.393	—	Core clock
CRY1	Amygdala	1.275	2.02 × 10⁻¹	.706	—	Core clock
BHLHE41	Frontal Cortex (BA9)	−1.000	3.17 × 10⁻¹	.801	—	Core clock
CLOCK	Frontal Cortex (BA9)	0.818	4.13 × 10⁻¹	.849	—	Core clock
BMAL1 (ARNTL)	Amygdala	−0.453	6.50 × 10⁻¹	.930	—	Core clock
PER2	Caudate	−0.206	8.37 × 10⁻¹	.973	—	Core clock
CSNK1E	Frontal Cortex (BA9)	−0.090	9.28 × 10⁻¹	.988	—	Core clock kinase
CRY2	Caudate	0.090	9.29 × 10⁻¹	.988	—	Core clock

**Table 14:** Top synaptic-pruning and MHC-related TWAS associations. Note. The pruning signal is dominated by the MHC-I antigen-processing axis (TAP2, PSMB8, HLA-A/B) and NF-κB inflammation (NFKB1, RELA), consistent with neuroimmune mechanisms linking atopy to brain phenotypes. TWAS = transcriptome-wide association study; FDR = false discovery rate; MHC = major histocompatibility complex; NF-κB = nuclear factor κB; TGF-β = transforming growth factor-beta; BMP = bone morphogenetic protein.

Gene	Best tissue	Z	p	FDR (q)	Sig.	Category
TAP2	Amygdala	6.645	3.02 × 10⁻¹¹	< .001	***	MHC-I antigen
ATG5	Hippocampus	5.472	4.44 × 10⁻⁸	< .001	***	Autophagy
PSMB8	Anterior Cing. Cortex	−4.898	9.70 × 10⁻⁷	< .001	***	Immunoproteasome
HLA-B	Hippocampus	−4.442	8.90 × 10⁻⁶	.001	**	MHC-I
NFKB1	Caudate	4.441	8.97 × 10⁻⁶	.001	**	NF-κB/inflammation
ROCK2	Frontal Cortex (BA9)	4.377	1.20 × 10⁻⁵	.002	**	Cytoskeletal pruning
RELA	Amygdala	4.117	3.84 × 10⁻⁵	.004	**	NF-κB p65
HLA-A	Amygdala	−3.951	7.77 × 10⁻⁵	.008	**	MHC-I
EFNA1	Nucleus Accumbens	−3.670	2.43 × 10⁻⁴	.018	*	Ephrin signalling
SEMA6A	Hippocampus	−3.458	5.44 × 10⁻⁴	.033	*	Semaphorin/guidance
SMAD4	Frontal Cortex (BA9)	3.169	1.53 × 10⁻³	.068	—	TGF-β signalling
CFL1	Anterior Cing. Cortex	3.131	1.74 × 10⁻³	.073	—	Actin dynamics
C4B	Anterior Cing. Cortex	3.000	2.70 × 10⁻³	.097	—	Complement
BMP4	Nucleus Accumbens	−2.983	2.86 × 10⁻³	.100	—	BMP signalling
MAP1LC3A	Frontal Cortex (BA9)	2.937	3.32 × 10⁻³	.111	—	Autophagy
PCDHGC3	Amygdala	2.786	5.34 × 10⁻³	.145	—	Protocadherin
IL1B	Anterior Cing. Cortex	−2.667	7.66 × 10⁻³	.180	—	Pro-inflammatory cytokine
NRP1	Caudate	−2.580	9.89 × 10⁻³	.206	—	Neuropilin/guidance
EFNB2	Hippocampus	2.540	1.11 × 10⁻²	.220	—	Ephrin signalling
ARPC2	Nucleus Accumbens	−2.491	1.27 × 10⁻²	.231	—	Arp2/3 complex

**Table 15:** Top glutamatergic, monoaminergic, and housekeeping TWAS associations. Note. TWAS = transcriptome-wide association study; FDR = false discovery rate; TCA = tricarboxylic-acid cycle; DA = dopamine; 5-HT = serotonin; PKC = protein kinase C; HAT = histone acetyl-transferase; AMPA = α-amino-3-hydroxy-5-methyl-4-isoxazolepropionic-acid receptor; NMDA = N-methyl-D-aspartate receptor; PSD = post-synaptic density. * q < 0.05. ** q < 0.01. *** q < 0.001.

Gene	Best tissue	Z	p	FDR (q)	Sig.	Gene Set
ACO2	Nucleus Accumbens	6.423	1.34 × 10⁻¹⁰	< .001	***	Housekeeping (TCA)
PNMT	Frontal Cortex (BA9)	−5.999	1.99 × 10⁻⁹	< .001	***	Monoamines (Epinephrine synth.)
HSPA1B	Frontal Cortex (BA9)	5.259	1.45 × 10⁻⁷	< .001	***	Housekeeping (Heat Shock)
PPP1R1B	Amygdala	4.859	1.18 × 10⁻⁶	< .001	***	Monoamines (DARPP-32)
RPS16	Nucleus Accumbens	4.791	1.66 × 10⁻⁶	< .001	***	Housekeeping (Ribosomal)
HTR1D	Nucleus Accumbens	−4.700	2.61 × 10⁻⁶	< .001	***	Monoamines (5-HT)
RPS11	Hippocampus	−4.611	4.02 × 10⁻⁶	< .001	***	Housekeeping (Ribosomal)
RPL13A	Frontal Cortex (BA9)	4.561	5.09 × 10⁻⁶	< .001	***	Housekeeping (Ribosomal)
FOSL1	Amygdala	4.345	1.39 × 10⁻⁵	.002	**	Glutamatergics (AP-1)
EP300	Frontal Cortex (BA9)	4.311	1.62 × 10⁻⁵	.002	**	Glutamatergics (HAT)
RPL5	Frontal Cortex (BA9)	4.281	1.86 × 10⁻⁵	.002	**	Housekeeping (Ribosomal)
CALML4	Anterior Cing. Cortex	−3.828	1.29 × 10⁻⁴	.011	*	Glutamatergics (Calmodulin-like)
HSPA2	Anterior Cing. Cortex	−3.691	2.24 × 10⁻⁴	.017	*	Housekeeping (Heat Shock)
PRKCB	Caudate	−3.641	2.72 × 10⁻⁴	.020	*	Glutamatergics (PKC-β)
NDUFA2	Anterior Cing. Cortex	3.514	4.41 × 10⁻⁴	.028	*	Housekeeping (Mito Cplx I)
HSPA1L	Nucleus Accumbens	3.411	6.48 × 10⁻⁴	.038	*	Housekeeping (Heat Shock)
GNG2	Anterior Cing. Cortex	3.167	1.54 × 10⁻³	.068	—	Monoamines (G-protein γ)
TSC1	Amygdala	−2.914	3.57 × 10⁻³	.116	—	Glutamatergics (mTOR)
GNG3	Frontal Cortex (BA9)	−2.838	4.54 × 10⁻³	.132	—	Monoamines (G-protein γ)
SLC22A3	Hippocampus	2.814	4.90 × 10⁻³	.138	—	Monoamines (OCT3)
GNB2	Frontal Cortex (BA9)	−2.678	7.41 × 10⁻³	.177	—	Monoamines (G-protein β)
HNMT	Nucleus Accumbens	2.671	7.56 × 10⁻³	.179	—	Monoamines (Histamine)
DRD2	Frontal Cortex (BA9)	−2.635	8.42 × 10⁻³	.190	—	Monoamines (D₂ receptor)
GRIA2	Frontal Cortex (BA9)	−2.622	8.73 × 10⁻³	.194	—	Glutamatergics (AMPA)
TSC2	Anterior Cing. Cortex	−2.612	9.00 × 10⁻³	.197	—	Glutamatergics (mTOR)
DLGAP1	Anterior Cing. Cortex	2.581	9.85 × 10⁻³	.206	—	Glutamatergics (PSD)
GRIN2B	Caudate	−2.000	4.55 × 10⁻²	.413	—	Glutamatergics (NMDA GluN2B)

**Table 16:** Summary of principal findings. Note. Prediction models: GTEx v8 MASHR eQTL (Zenodo DOI: 10.5281/zenodo.3518299). GWAS: Allergic-disease meta-analysis EBI-a-GCST005038 (N = 360,838). Brain transcriptomic convergence implicates MHC-I antigen processing, NF-κB inflammation, and proteasome-linked circadian regulators as candidate mediators of atopy-brain comorbidity. GWAS = genome-wide association study; MHC = major histocompatibility complex; NF-κB = nuclear factor κB; FDR = false discovery rate.

Domain	Finding
Strongest single-gene hit	TAP2 in Amygdala (Z = 6.65, p = 3.02 × 10⁻¹¹) — MHC-class-I antigen transporter, linking atopic-disease genetic risk to neuroimmune transcription in limbic circuitry.
Neuroimmune/pruning axis	The pruning set reached the largest raw number of FDR hits (10/178), dominated by MHC-I (TAP2, HLA-A/B, PSMB8) and NF-κB (NFKB1, RELA) signalling, plus autophagy regulators ATG5 and MAP1LC3A.
Circadian signal (brain-specific pattern)	Reactome Circadian Clock showed nominal enrichment (1.12×; p = .019) driven by proteasome subunits (PSMD3, PSMB7, PSMA6, PSMA4) and NR1D1/BTRC, rather than canonical CLOCK-BMAL1-PER-CRY oscillator genes.
Glutamatergic signal	Nominally enriched (1.07×; p = .013) with four FDR hits: FOSL1, EP300, CALML4, PRKCB — highlighting activity-dependent transcription and calcium signalling.
Monoaminergic signal	Three FDR hits (PNMT, PPP1R1B/DARPP-32, HTR1D); overall set not enriched (1.02×; p = .766), suggesting hits are specific rather than pathway-wide.
Housekeeping controls	Nine FDR-significant housekeeping genes (ACO2, HSPA1B/2/1L, RPS11/16, RPL5/13A, NDUFA2) indicate broad transcriptomic perturbation, likely reflecting very high GWAS power (N = 360,838).
Target-vs-control comparison	Target sets did not exceed control enrichment in the brain (target 1.02× vs. control 1.05×), in contrast to the peripheral-tissue analysis, where circadian targets showed specific enrichment.
Global numbers	68,860 gene–tissue tests across 6 brain tissues and 16,426 unique genes; 1,359 FDR-significant associations (535 unique genes); 348 Bonferroni-significant (149 unique genes).

Appendix C: Exhaustive GSEA pipeline: Results tables

ATOP (Allergy) and OCD GWAS - Circadian, Glial, Clonal-Hematopoiesis, and Control Gene Sets

Permutations = 1,000; Weighted = True; Set size 5-500; Significance α = 0.05; Random seed = 42.

**Table 17:** Standard GSEA results for ATOP and OCD across all gene sets. Note. Results sorted by permutation p-value. GSEA = gene set enrichment analysis; ATOP = atopy/atopic diseases; OCD = obsessive-compulsive disorder; ES = enrichment score; NES = normalised enrichment score; FDR = false discovery rate; n Hits = number of gene-set members in the ranked list; LE = leading-edge size. Subset names include trailing "_ALL" for combined versions. * p < 0.05. ** p < 0.01.

Sample	Gene set	ES	NES	p	FDR	n Hits	LE
ATOP	Reactome_Circadian_Clock	0.479	1.523	.001**	.017	106	38
ATOP	Reactome_Circadian_Clock_ALL	0.479	1.502	.001**	.022	106	38
OCD	GOBP_Regulation_Circadian_Rhythm	0.442	1.607	.001**	.009	111	40
OCD	GOBP_Regulation_Circadian_Rhythm_ALL	0.442	1.589	.001**	.011	111	40
OCD	Adult_Astrocyte_ALL	-0.374	-1.585	.008**	.237	36	15
OCD	Adult_Astrocyte	-0.374	-1.594	.008**	.223	36	15
ATOP	Clonal_Hematopoiesis_ALL	0.491	1.489	.008**	.024	64	35
ATOP	Clonal_Hematopoiesis	0.491	1.483	.008**	.026	64	35
OCD	Reactome_Circadian_Clock_ALL	0.402	1.442	.009**	.045	106	38
OCD	Reactome_Circadian_Clock	0.402	1.453	.010*	.041	106	38
OCD	Control_Housekeeping_Heat_Shock_Proteins	0.577	1.545	.016*	.018	17	12
ATOP	Control_Housekeeping_Heat_Shock_Proteins	0.613	1.559	.023*	.012	17	5
OCD	Control_Monoamine_Downstream_Signaling	0.509	1.426	.058	.052	20	13
OCD	Control_Housekeeping_ALL	0.340	1.261	.062	.166	158	42
OCD	Control_Monoamine_Vesicular_Machinery	0.574	1.370	.088	.077	11	5
OCD	WP_Circadian_Rhythm_Genes_ALL	0.321	1.215	.089	.221	192	67
OCD	WP_Circadian_Rhythm_Genes	0.321	1.202	.098	.237	192	67
OCD	Adult_Oligodendrocyte	0.434	1.324	.105	.109	33	18
OCD	KEGG_Circadian_Rhythm_Mammal_ALL	0.539	1.358	.106	.085	12	6
OCD	Adult_Oligodendrocyte_ALL	0.434	1.323	.106	.110	33	18
OCD	KEGG_Circadian_Rhythm_Mammal	0.539	1.322	.126	.110	12	6
OCD	Control_Housekeeping_Glycolysis	0.475	1.331	.126	.104	22	5
ATOP	Control_Housekeeping_Ribosomal_Small_Subunit	0.494	1.279	.151	.132	20	8
ATOP	GOBP_Regulation_Circadian_Rhythm_ALL	0.367	1.158	.179	.277	111	23
ATOP	Control_Monoamine_Dopamine_Transport_Signaling	0.596	1.255	.184	.156	8	2
ATOP	Control_Monoamine_Dopamine_Receptors	-0.578	-1.273	.191	1.000	5	4
ATOP	GOBP_Regulation_Circadian_Rhythm	0.367	1.150	.198	.288	111	23
OCD	Control_Monoamine_Trace_Amine_Receptors	-0.508	-1.226	.203	1.000	6	6
OCD	Control_Housekeeping_Ribosomal_Large_Subunit	0.399	1.134	.300	.338	23	8
ATOP	Control_Housekeeping_TCA_Cycle	0.450	1.138	.307	.307	18	3
OCD	Control_Housekeeping_Mitochondrial_Complex_IV	0.430	1.136	.317	.334	15	9
ATOP	Control_Monoamine_Vesicular_Machinery	0.499	1.145	.327	.297	11	7
ATOP	Adult_Microglia_ALL	0.421	1.125	.327	.329	24	12
ATOP	Adult_Microglia	0.421	1.119	.351	.339	24	12
OCD	Control_Housekeeping_Ribosomal_Small_Subunit	0.383	1.075	.371	.443	20	7
OCD	Control_Monoamine_ALL	0.294	1.046	.407	.497	96	36
OCD	Control_Housekeeping_TCA_Cycle	0.381	1.034	.422	.521	18	12
OCD	Control_Housekeeping_Mitochondrial_Complex_I	-0.286	-1.020	.425	1.000	18	6
OCD	Control_Monoamine_Histamine_System	0.500	1.046	.425	.496	7	2
ATOP	Control_Monoamine_Histamine_System	0.504	1.036	.446	.479	7	4
ATOP	KEGG_Circadian_Rhythm_Mammal	0.432	1.009	.461	.530	12	3
ATOP	Control_Monoamine_Norepinephrine_Receptors	-0.350	-0.978	.461	1.000	9	2
ATOP	KEGG_Circadian_Rhythm_Mammal_ALL	0.432	1.016	.465	.518	12	3
ATOP	Control_Housekeeping_ALL	0.309	0.990	.522	.563	158	61
ATOP	WP_Circadian_Rhythm_Genes	0.306	0.984	.541	.575	192	39
OCD	Control_Monoamine_Norepinephrine_Receptors	-0.332	-0.928	.544	1.000	9	5
ATOP	WP_Circadian_Rhythm_Genes_ALL	0.306	0.986	.562	.573	192	39
ATOP	Control_Housekeeping_Ribosomal_Large_Subunit	0.361	0.955	.564	.626	23	10
ATOP	Adult_Oligodendrocyte	0.326	0.922	.595	.683	33	7
ATOP	Adult_Oligodendrocyte_ALL	0.326	0.922	.606	.683	33	7
OCD	Control_Housekeeping_Cytoskeletal_Actin	0.386	0.906	.609	.767	10	2
ATOP	Control_Monoamine_Trace_Amine_Receptors	0.457	0.908	.627	.705	6	2
ATOP	Control_Monoamine_ALL	0.303	0.945	.631	.645	96	39
OCD	Control_Monoamine_Dopamine_Receptors	0.464	0.895	.643	.788	5	2
ATOP	Control_Monoamine_Downstream_Signaling	0.323	0.842	.691	.807	20	9
OCD	Control_Monoamine_Dopamine_Synthesis_Metabolism	0.380	0.835	.701	.891	8	3
OCD	Control_Monoamine_Serotonin_Receptors	-0.240	-0.821	.752	1.000	16	7
ATOP	Adult_Astrocyte	0.285	0.805	.774	.857	36	12
ATOP	Adult_Astrocyte_ALL	0.285	0.803	.774	.861	36	12
ATOP	Control_Housekeeping_Cytoskeletal_Actin	0.333	0.747	.783	.923	10	1
OCD	Control_Housekeeping_Cytoskeletal_Tubulin	0.279	0.728	.843	1.000	15	4
ATOP	Control_Housekeeping_Cytoskeletal_Tubulin	-0.215	-0.758	.846	1.000	15	15
OCD	Clonal_Hematopoiesis_ALL	0.221	0.745	.872	1.000	64	8
OCD	Clonal_Hematopoiesis	0.221	0.750	.873	1.000	64	8
ATOP	Control_Monoamine_Dopamine_Synthesis_Metabolism	0.314	0.665	.882	.998	8	4
OCD	Adult_Microglia	0.221	0.640	.926	1.000	24	6
OCD	Adult_Microglia_ALL	0.221	0.647	.926	1.000	24	6
ATOP	Control_Housekeeping_Mitochondrial_Complex_I	0.237	0.601	.928	1.000	18	3
ATOP	Control_Monoamine_Serotonin_Receptors	0.225	0.561	.950	1.000	16	6
ATOP	Control_Housekeeping_Mitochondrial_Complex_IV	0.222	0.537	.965	1.000	15	6
ATOP	Control_Housekeeping_Glycolysis	0.173	0.464	.989	1.000	22	12
OCD	Control_Monoamine_Dopamine_Transport_Signaling	0.210	0.462	.994	1.000	8	3

**Table 18:** Cross-sample comparison of 10 major gene sets (ATOP vs. OCD). Note. Side-by-side comparison of the 10 major gene sets (combined "_ALL" versions) across ATOP (allergy) and OCD GWAS. n Hits are identical by gene set, but gene-ranking universe sizes differ slightly (ATOP, n = 18,270; OCD, n = 18,279). ** p < 0.01. ATOP = atopy/atopic diseases; OCD = obsessive-compulsive disorder; ES = enrichment score; NES = normalised enrichment score; FDR = false discovery rate; n Hits = number of gene-set members in the ranked list.

Gene set	Sample	ES	NES	p	FDR	n Hits	Sample	ES	NES	p	FDR
Reactome_Circadian_Clock_ALL	ATOP	0.479	1.502	.001**	.022	106	OCD	0.402	1.442	.009**	.045
KEGG_Circadian_Rhythm_Mammal_ALL	ATOP	0.432	1.016	.465	.518	12	OCD	0.539	1.358	.106	.085
WP_Circadian_Rhythm_Genes_ALL	ATOP	0.306	0.986	.562	.573	192	OCD	0.321	1.215	.089	.221
GOBP_Regulation_Circadian_Rhythm_ALL	ATOP	0.367	1.158	.179	.277	111	OCD	0.442	1.589	.001**	.011
Adult_Microglia_ALL	ATOP	0.421	1.125	.327	.329	24	OCD	0.221	0.647	.926	1.000
Adult_Astrocyte_ALL	ATOP	0.285	0.803	.774	.861	36	OCD	-0.374	-1.585	.008**	.237
Adult_Oligodendrocyte_ALL	ATOP	0.326	0.922	.606	.683	33	OCD	0.434	1.323	.106	.110
Clonal_Hematopoiesis_ALL	ATOP	0.491	1.489	.008**	.024	64	OCD	0.221	0.745	.872	1.000
Control_Monoamine_ALL	ATOP	0.303	0.945	.631	.645	96	OCD	0.294	1.046	.407	.497
Control_Housekeeping_ALL	ATOP	0.309	0.990	.522	.563	158	OCD	0.340	1.261	.062	.166

**Table 19:** Exhaustive differential GSEA (DGSEA): All pairwise comparisons among 10 major gene sets. Note. All 45 pairwise DGSEA comparisons × 2 samples (N = 90). Set labels abbreviated for space (e.g., Reactome_Circadian = Reactome_Circadian_Clock_ALL; GOBP_CircReg = GOBP_Regulation_Circadian_Rhythm_ALL; Clonal_Hemato = Clonal_Hematopoiesis_ALL; Ctrl_Monoamine / Ctrl_Housekeeping = combined "_ALL" control sets). ATOP = atopy/atopic diseases; GSEA = gene set enrichment analysis; ES = enrichment score; NES = normalised enrichment score; ΔES = ES_A_ − ES_B_; ΔNES = NES_A_ − NES_B_; pΔ is the permutation p-value for the difference. * p < 0.05. ** p < 0.01.

Sample	Set A	Set B	ES_A_	ES_B_	ΔES	NES_A_	NES_B_	ΔNES	p_Δ_
ATOP	Reactome_Circadian	KEGG_Circadian	0.479	0.432	+0.047	1.502	1.016	+0.486	.274
OCD	Reactome_Circadian	KEGG_Circadian	0.402	0.539	-0.136	1.442	1.358	+0.084	.369
ATOP	Reactome_Circadian	WP_Circadian	0.479	0.306	+0.173	1.502	0.986	+0.516	.013
OCD	Reactome_Circadian	WP_Circadian	0.402	0.321	+0.081	1.442	1.215	+0.227	.149
ATOP	Reactome_Circadian	GOBP_CircReg	0.479	0.367	+0.112	1.502	1.158	+0.344	.089
OCD	Reactome_Circadian	GOBP_CircReg	0.402	0.442	-0.040	1.442	1.589	-0.147	.299
ATOP	Reactome_Circadian	Adult_Microglia	0.479	0.421	+0.058	1.502	1.125	+0.377	.239
OCD	Reactome_Circadian	Adult_Microglia	0.402	0.221	+0.182	1.442	0.647	+0.795	.179
ATOP	Reactome_Circadian	Adult_Astrocyte	0.479	0.285	+0.194	1.502	0.803	+0.699	.061
OCD	Reactome_Circadian	Adult_Astrocyte	0.402	-0.374	+0.776	1.442	-1.585	+3.027	.001**
ATOP	Reactome_Circadian	Adult_Oligo	0.479	0.326	+0.153	1.502	0.922	+0.579	.096
OCD	Reactome_Circadian	Adult_Oligo	0.402	0.434	-0.032	1.442	1.323	+0.119	.454
ATOP	Reactome_Circadian	Clonal_Hemato	0.479	0.491	-0.012	1.502	1.489	+0.013	.512
OCD	Reactome_Circadian	Clonal_Hemato	0.402	0.221	+0.182	1.442	0.745	+0.697	.070
ATOP	Reactome_Circadian	Ctrl_Monoamine	0.479	0.303	+0.176	1.502	0.945	+0.557	.021
OCD	Reactome_Circadian	Ctrl_Monoamine	0.402	0.294	+0.109	1.442	1.046	+0.396	.110
ATOP	Reactome_Circadian	Ctrl_Housekeeping	0.479	0.309	+0.170	1.502	0.990	+0.511	.007**
OCD	Reactome_Circadian	Ctrl_Housekeeping	0.402	0.340	+0.062	1.442	1.261	+0.181	.220
ATOP	KEGG_Circadian	WP_Circadian	0.432	0.306	+0.126	1.016	0.986	+0.030	.388
OCD	KEGG_Circadian	WP_Circadian	0.539	0.321	+0.218	1.358	1.215	+0.143	.208
ATOP	KEGG_Circadian	GOBP_CircReg	0.432	0.367	+0.065	1.016	1.158	-0.142	.528
OCD	KEGG_Circadian	GOBP_CircReg	0.539	0.442	+0.097	1.358	1.589	-0.231	.470
ATOP	KEGG_Circadian	Adult_Microglia	0.432	0.421	+0.011	1.016	1.125	-0.109	.511
OCD	KEGG_Circadian	Adult_Microglia	0.539	0.221	+0.318	1.358	0.647	+0.711	.147
ATOP	KEGG_Circadian	Adult_Astrocyte	0.432	0.285	+0.147	1.016	0.803	+0.213	.289
OCD	KEGG_Circadian	Adult_Astrocyte	0.539	-0.374	+0.913	1.358	-1.585	+2.943	.001**
ATOP	KEGG_Circadian	Adult_Oligo	0.432	0.326	+0.107	1.016	0.922	+0.093	.369
OCD	KEGG_Circadian	Adult_Oligo	0.539	0.434	+0.104	1.358	1.323	+0.035	.380
ATOP	KEGG_Circadian	Clonal_Hemato	0.432	0.491	-0.059	1.016	1.489	-0.473	.284
OCD	KEGG_Circadian	Clonal_Hemato	0.539	0.221	+0.318	1.358	0.745	+0.613	.097
ATOP	KEGG_Circadian	Ctrl_Monoamine	0.432	0.303	+0.129	1.016	0.945	+0.071	.354
OCD	KEGG_Circadian	Ctrl_Monoamine	0.539	0.294	+0.245	1.358	1.046	+0.312	.144
ATOP	KEGG_Circadian	Ctrl_Housekeeping	0.432	0.309	+0.124	1.016	0.990	+0.025	.374
OCD	KEGG_Circadian	Ctrl_Housekeeping	0.539	0.340	+0.199	1.358	1.261	+0.097	.218
ATOP	WP_Circadian	GOBP_CircReg	0.306	0.367	-0.061	0.986	1.158	-0.172	.216
OCD	WP_Circadian	GOBP_CircReg	0.321	0.442	-0.121	1.215	1.589	-0.374	.050
ATOP	WP_Circadian	Adult_Microglia	0.306	0.421	-0.114	0.986	1.125	-0.139	.293
OCD	WP_Circadian	Adult_Microglia	0.321	0.221	+0.100	1.215	0.647	+0.568	.187
ATOP	WP_Circadian	Adult_Astrocyte	0.306	0.285	+0.021	0.986	0.803	+0.183	.311
OCD	WP_Circadian	Adult_Astrocyte	0.321	-0.374	+0.695	1.215	-1.585	+2.800	.001**
ATOP	WP_Circadian	Adult_Oligo	0.306	0.326	-0.019	0.986	0.922	+0.063	.538
OCD	WP_Circadian	Adult_Oligo	0.321	0.434	-0.113	1.215	1.323	-0.108	.246
ATOP	WP_Circadian	Clonal_Hemato	0.306	0.491	-0.185	0.986	1.489	-0.503	.034
OCD	WP_Circadian	Clonal_Hemato	0.321	0.221	+0.100	1.215	0.745	+0.470	.107
ATOP	WP_Circadian	Ctrl_Monoamine	0.306	0.303	+0.003	0.986	0.945	+0.041	.414
OCD	WP_Circadian	Ctrl_Monoamine	0.321	0.294	+0.027	1.215	1.046	+0.169	.310
ATOP	WP_Circadian	Ctrl_Housekeeping	0.306	0.309	-0.002	0.986	0.990	-0.005	.493
OCD	WP_Circadian	Ctrl_Housekeeping	0.321	0.340	-0.019	1.215	1.261	-0.046	.388
ATOP	GOBP_CircReg	Adult_Microglia	0.367	0.421	-0.053	1.158	1.125	+0.033	.453
OCD	GOBP_CircReg	Adult_Microglia	0.442	0.221	+0.222	1.589	0.647	+0.942	.167
ATOP	GOBP_CircReg	Adult_Astrocyte	0.367	0.285	+0.082	1.158	0.803	+0.355	.200
OCD	GOBP_CircReg	Adult_Astrocyte	0.442	-0.374	+0.816	1.589	-1.585	+3.174	.001**
ATOP	GOBP_CircReg	Adult_Oligo	0.367	0.326	+0.042	1.158	0.922	+0.235	.280
OCD	GOBP_CircReg	Adult_Oligo	0.442	0.434	+0.008	1.589	1.323	+0.266	.382
ATOP	GOBP_CircReg	Clonal_Hemato	0.367	0.491	-0.124	1.158	1.489	-0.331	.105
OCD	GOBP_CircReg	Clonal_Hemato	0.442	0.221	+0.221	1.589	0.745	+0.844	.070
ATOP	GOBP_CircReg	Ctrl_Monoamine	0.367	0.303	+0.064	1.158	0.945	+0.213	.203
OCD	GOBP_CircReg	Ctrl_Monoamine	0.442	0.294	+0.148	1.589	1.046	+0.543	.056
ATOP	GOBP_CircReg	Ctrl_Housekeeping	0.367	0.309	+0.059	1.158	0.990	+0.168	.226
OCD	GOBP_CircReg	Ctrl_Housekeeping	0.442	0.340	+0.102	1.589	1.261	+0.328	.107
ATOP	Adult_Microglia	Adult_Astrocyte	0.421	0.285	+0.136	1.125	0.803	+0.322	.209
OCD	Adult_Microglia	Adult_Astrocyte	0.221	-0.374	+0.595	0.647	-1.585	+2.232	.055
ATOP	Adult_Microglia	Adult_Oligo	0.421	0.326	+0.095	1.125	0.922	+0.203	.313
OCD	Adult_Microglia	Adult_Oligo	0.221	0.434	-0.214	0.647	1.323	-0.676	.167
ATOP	Adult_Microglia	Clonal_Hemato	0.421	0.491	-0.070	1.125	1.489	-0.364	.257
OCD	Adult_Microglia	Clonal_Hemato	0.221	0.221	-0.000	0.647	0.745	-0.098	.423
ATOP	Adult_Microglia	Ctrl_Monoamine	0.421	0.303	+0.118	1.125	0.945	+0.180	.251
OCD	Adult_Microglia	Ctrl_Monoamine	0.221	0.294	-0.073	0.647	1.046	-0.399	.243
ATOP	Adult_Microglia	Ctrl_Housekeeping	0.421	0.309	+0.112	1.125	0.990	+0.135	.313
OCD	Adult_Microglia	Ctrl_Housekeeping	0.221	0.340	-0.119	0.647	1.261	-0.614	.176
ATOP	Adult_Astrocyte	Adult_Oligo	0.285	0.326	-0.041	0.803	0.922	-0.120	.388
OCD	Adult_Astrocyte	Adult_Oligo	-0.374	0.434	-0.808	-1.585	1.323	-2.908	.001**
ATOP	Adult_Astrocyte	Clonal_Hemato	0.285	0.491	-0.206	0.803	1.489	-0.686	.073
OCD	Adult_Astrocyte	Clonal_Hemato	-0.374	0.221	-0.595	-1.585	0.745	-2.330	.029
ATOP	Adult_Astrocyte	Ctrl_Monoamine	0.285	0.303	-0.018	0.803	0.945	-0.142	.355
OCD	Adult_Astrocyte	Ctrl_Monoamine	-0.374	0.294	-0.668	-1.585	1.046	-2.631	.003**
ATOP	Adult_Astrocyte	Ctrl_Housekeeping	0.285	0.309	-0.023	0.803	0.990	-0.188	.285
OCD	Adult_Astrocyte	Ctrl_Housekeeping	-0.374	0.340	-0.714	-1.585	1.261	-2.846	.001**
ATOP	Adult_Oligo	Clonal_Hemato	0.326	0.491	-0.166	0.922	1.489	-0.566	.099
OCD	Adult_Oligo	Clonal_Hemato	0.434	0.221	+0.214	1.323	0.745	+0.578	.081
ATOP	Adult_Oligo	Ctrl_Monoamine	0.326	0.303	+0.023	0.922	0.945	-0.022	.551
OCD	Adult_Oligo	Ctrl_Monoamine	0.434	0.294	+0.141	1.323	1.046	+0.277	.172
ATOP	Adult_Oligo	Ctrl_Housekeeping	0.326	0.309	+0.017	0.922	0.990	-0.068	.535
OCD	Adult_Oligo	Ctrl_Housekeeping	0.434	0.340	+0.094	1.323	1.261	+0.062	.298
ATOP	Clonal_Hemato	Ctrl_Monoamine	0.491	0.303	+0.188	1.489	0.945	+0.544	.028
OCD	Clonal_Hemato	Ctrl_Monoamine	0.221	0.294	-0.073	0.745	1.046	-0.301	.202
ATOP	Clonal_Hemato	Ctrl_Housekeeping	0.491	0.309	+0.183	1.489	0.990	+0.498	.020
OCD	Clonal_Hemato	Ctrl_Housekeeping	0.221	0.340	-0.119	0.745	1.261	-0.516	.102
ATOP	Ctrl_Monoamine	Ctrl_Housekeeping	0.303	0.309	-0.006	0.945	0.990	-0.046	.432
OCD	Ctrl_Monoamine	Ctrl_Housekeeping	0.294	0.340	-0.046	1.046	1.261	-0.215	.253

**Table 20:** Summary of analysis and key findings. Note. Permutations = 1,000; weighted statistic; α = 0.05. ns = nonsignificant. The suffix "_ALL" denotes a combined gene set formed by the union across subset components. Differential GSEA (DGSEA) tests whether the enrichment of gene set A differs significantly from that of gene set B within the same ranked gene list. GSEA = gene set enrichment analysis; GWAS = genome-wide association study; ATOP = atopy/atopic diseases; OCD = obsessive-compulsive disorder; NES = normalised enrichment score.

Metric	ATOP (Allergy GWAS)	OCD GWAS
Genes ranked	18,270	18,279
Gene sets tested (size 5–500)	36	36
Significant at p < .05	5 (13.9%)	7 (19.4%)
Most significant positive enrichment	Reactome_Circadian_Clock (NES = 1.523, p = .001)	GOBP_Regulation_Circadian_Rhythm (NES = 1.607, p = .001)
Most significant negative enrichment	Control_Monoamine_Dopamine_Receptors (NES = −1.273, p = .191, ns)	Adult_Astrocyte (NES = −1.594, p = .008)
Other significant circadian hits	Reactome_Circadian_Clock_ALL (p = .001)	Reactome_Circadian_Clock (p = .010); Reactome_Circadian_Clock_ALL (p = .009)
Unique sample-specific finding	Clonal_Hematopoiesis enrichment (NES = 1.489, p = .008)	Astrocyte marker depletion (NES = −1.585, p = .008)
Shared finding (both samples)	Reactome_Circadian_Clock (positive; both p < .01); Heat Shock Proteins (positive; both p < .05)	
Significant DGSEA contrasts (p_Δ_ < .05)	6 of 45 (e.g., Reactome_Circadian > Ctrl_Housekeeping, p = .007; Clonal_Hemato > Ctrl_Housekeeping, p = .020)	7 of 45 (all involving Adult_Astrocyte being lower than circadian/other sets, p ≤ .003)
